# A comparative study of microbial community and dynamics of *Asaia* in the brown planthopper from susceptible and resistant rice varieties

**DOI:** 10.1186/s12866-019-1512-9

**Published:** 2019-06-24

**Authors:** Abhishek Ojha, Wenqing Zhang

**Affiliations:** 1 State Key Laboratory of Biocontrol and School of Life Sciences, Sun Yat-sen University, Guangzhou, 510275 Guangdong China; 2 State Key Laboratory of Tea Plant Biology and Utilization, Anhui Agricultural University, Hefei, 230036 Anhui China

**Keywords:** Operational taxonomic units, Diversity, Microbiome, 16S rRNA, Macropterous and brachypterous BPH, Insect-plant interaction

## Abstract

**Background:**

The brown planthopper (BPH) is likely the most destructive, piercing and sucking monophagous insect pest of rice that causes substantial economic losses to farmers. Although yeast**-**like symbionts (YLS) and virus transmission have been observed in the BPH, the bacterial population inhabiting the BPH has received minimal research attention. Labelling BPH**-**associated bacterial species may shed light on BPH biology and the interaction between the BPH and rice to provide novel approaches for the efficient control of this insect pest.

**Results:**

We examined RNA**-**seq results to identify bacterial populations present in different generations of BPHs maintained on susceptible or resistant rice varieties. Overall, 87 operational taxonomic units (OTUs) were determined from the BPH**-**F0, F6 and F16 generations. These OTUs had Shannon and Simpson index values of 0.37–0.6 and 0.56–1.19, respectively. The evenness values of 0.7–1.00 showed the vastness of the bacterial diversity recovered from the BPH samples. The results showed high species diversity in the BPHs collected from susceptible rice and a high number of members of unclassified bacteria in the BPHs isolated from resistant rice. We noticed that Proteobacteria OTUs were predominant across all samples. Furthermore, PCR data of *Asaia* species showed variable DNA amplification across the BPH samples collected from susceptible or resistant varieties. The identification of *Asaia* in BPH eggs and BPH**-**egg**-**infected rice revealed its influence on the interaction between the BPH egg and rice.

**Conclusions:**

The BPHs had clear differences in their microbiomes and in their ability to feed on different rice hosts. These variations could have an essential impact on host adaptation and interaction. These results provide a better understanding of the bacterial diversity and interaction of the microbiome of different generations of BPHs. Furthermore, PCR data of *Asaia* sp. variation across the BPH samples (isolated from different host genotypes selected from the field and laboratory, including BPH eggs and egg-infected rice tissues), suggest that *Asaia* could be an important member of the insect microbiome involved in adaptation, its interaction with rice and, most importantly, as a paratransgenic tool for insect control.

**Electronic supplementary material:**

The online version of this article (10.1186/s12866-019-1512-9) contains supplementary material, which is available to authorized users.

## Background

Microbes and insects have co**-**evolved for several hundred million years, and these associations are often reflected by extensive symbiotic relationships between partners. Microbes have been found to be involved in various phenomena of their host insects [1–4]. Previous reports have suggested that two planthopper species, *Laodelphax striatellus* and *Sogatella furcifera*, harbour microbes that can be transferred horizontally between different species of insects and strongly influence their hosts’ immune response, sexual reproduction and cytoplasmic incompatibility [4–6]. The role of microbes in insects using various molecular techniques has unveiled the unexplored world of microbe-insect interactions. However, further investigations are required to derive fruitful conclusions.

The brown planthopper, BPH, *Nilaparvata lugens* (Stål) (Hemiptera: Delphacidae) is a major monophagous agricultural pest of rice crops in the temperate and tropical regions of Asia [7]. The BPH has two winged forms (macropterous and brachypterous) at the adult stage. The macropterous (long-winged) BPH has long**-**distance migration behaviour [8] and infests rice fields, while the brachypterous (short-winged) BPH is known for laying many eggs and producing numerous offspring in rice hosts [9, 10] and can also quickly overcome resistance genes of its rice host by developing new virulence factors [11–13]. These characteristics make the BPH difficult to forecast and control. The BPH infests rice at all stages of plant growth. As a result of feeding by both nymphs and adults at the base of tillers, plants become yellow and ultimately die. This symptom of crop damage is known as “hopper burn” “(http://www.rkmp.co.in/content/brown**-**plant**-**hopper**-**bph)”.

Furthermore, recent reports revealed that endosymbionts of insects [14], which show an important role in uric acid metabolism [15], might also play a major role in insect**-**plant interactions [16, 17], thereby forming an integral part of BPH adaption to its rice host. However, while BPH**-**associated microbiomes and their modes of action are known, their stability is not clear. Here, we examine and classify the bacterial population of the BPH in an attempt to explain this interaction and gain important insights into this interaction to develop a new approach to manage this serious insect pest.

## Results

### Taxonomic diversity index

**Fig. 1:**
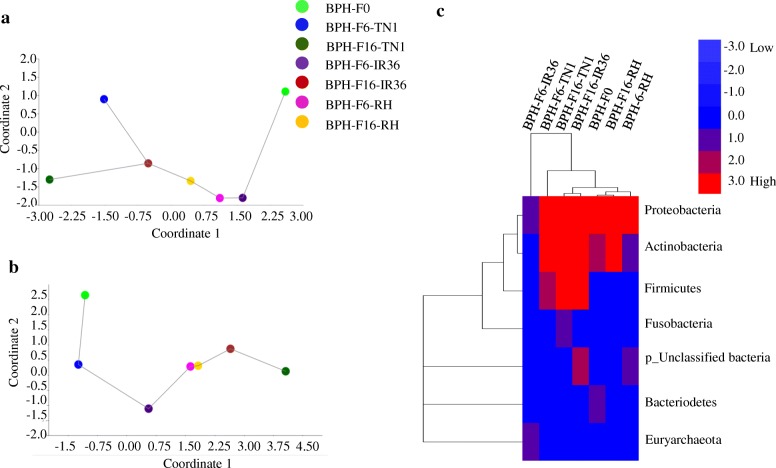
Evaluation of bacterial diversity in the BPH samples. Bacterial diversity in the BPH samples using principal coordinate analysis (PCoA); (**a**) similar**-** and (**b**) distance**-**PCoA plots show the quantitative evaluation of the bacterial diversity between the BPH samples, and (**c**) the heat map shows hierarchical clustering of OTUs. The scale bar represents the colour saturation gradient based on bacterial OTUs

The taxonomic diversity (normal distribution and alpha**-** and beta**-**diversity) of the bacterial population was analysed using RNA**-**seq data from different generations of adult female BPH samples. Normal distributions of the bacterial population within the BPH samples were calculated using the Shapiro**-**Wilk W and Anderson**-**Darling A test (see Additional file 1). The Shapiro**-**Wilk W index of normality ranged from 0.6 to 0.820, with a *P***-**value of 0.0002 to 0.064. The observed Anderson-Darling A probability distribution ranged from 0.565 to 1.423, with a *P***-**value of 0.0003 to 0.088 (see Additional file 1). Alpha**-**diversity indices (Shannon, Simpson, Evenness, Brillouin, Fisher alpha, Berger**-**Parker and Chao1) of the bacterial population were notably distinct within the BPH samples (Table 1). Shannon diversity values varied from 0.37 to 0.64, Simpson diversity values varied from 0.56 to 1.19, Evenness diversity values varied from 0.70 to 1.00 and Brillouin information diversity values varied from 0.34 to 0.99 (Table 1). The Fisher alpha and Berger**-**Parker species richness values ranged from 0.00 to 2.38 and 0.40 to 0.75, respectively (Table 1). The species richness (Chao1) estimator indicated that species richness ranged from 2 to 4, with the highest value in BPH**-**F16**-**TN1 and BPH**-**F16**-**IR36 among the BPH samples (Table 1). Notably, the BPH**-**F16**-**TN1 sample had more operational taxonomic units (OTUs) than the other BPH samples (Table 1). PAST (Palaeontological Statistics, version 3) software was used to analyse the beta diversity of the bacterial population between the BPH samples (as shown by PCoA). PCoA (principal coordinate analysis; similarity and distance) was calculated using eigenvalues and eigenvectors (coordinates). The similarity/distance eigenvalues were raised to the power of *C* (the transformation exponent) before Eigen analysis, and the standard value was *C* = 2. The “Eigenvalue scaling” option scale was used for each axis using the square root of the eigenvalue, and the minimal spanning tree option was based on the selected similarity or distance index. Similarity**-** and distance**-**PCoA estimated the presence or absence of bacterial species between the BPH (BPH**-**F0, BPH**-**F6**-**TN1, BHP**-**F16**-**TN1, BPH**-**F6**-**IR36, BPH**-**F16**-**IR36, BPH**-**F6**-**RH, and BPH**-**F16**-**RH) samples (Fig. 1). Similarity**-** (Fig. 1a) and distance**-**PCoA (Fig. 1b) plots revealed the bacterial diversity in the samples and noticeably separated the BPH**-**F0, BPH**-**F6**-**TN1, BHP**-**F16**-**TN1, BPH**-**F6**-**IR36, BPH**-**F16**-**IR36, BPH**-**F6**-**RH, and BPH**-**F16**-**RH samples. Evaluation of the PCoA data of pair**-**wise BPH sample comparisons was performed using Bonferroni correction (Table 2). Eight pair**-**wise BPH sample comparisons (BPH**-**F0 and BPH**-**F6**-**TN1, BPH**-**F0 and BPH**-**F6**-**IR36, BPH**-**F6**-**TN1 and BPH**-**F6**-**IR36, BPH**-**F6**-**TN1 and BPH**-**F6**-**RH, BPH**-**F16**-**TN1 and BPH**-**F6**-**IR36, BPH**-**F6**-**IR36 and BPH**-**F16**-**IR36, BPH**-**F6**-**IR36 and BPH**-**F6**-**RH, and BPH**-**F6**-**IR36 and BPH**-**F16**-**RH) had a Bonferroni corrected *P***-**value of 1.0, and the remaining 34 pair**-**wise sample comparisons had Bonferroni corrected *P***-**values in the range of 0.987 to 0.00 (Table 2). Furthermore, the Whittaker indices for similarities between bacterial communities of the BPH samples ranged from 0.143–0.666 (Table 3).

**Table 1:** Alpha-diversity indices and the estimated richness of the bacterial population in the BPH samples

S. No.	Sample name	Shannon	Simpson	Evenness	Brillouin	Fisher_alpha	Berger-Parker	Chao1	Observed Species
1.	BPH-F0	0.46	0.80	0.74	0.58	1.45	0.70	3	10
2.	BPH-F6-TN1	0.64	1.05	0.95	0.80	1.45	0.40	3	10
3.	BPH-F16-TN1	0.56	1.03	0.70	0.86	1.34	0.60	4	25
4.	BPH-F6-IR36	0.50	0.69	1.00	0.34	0.00	0.50	3	2
5.	BPH-F16-IR36	0.64	1.19	0.82	0.99	1.43	0.50	4	22
6.	BPH-F6-RH	0.50	0.86	0.79	0.56	2.38	0.66	4	6
7	BPH-F16-RH	0.37	0.56	0.87	0.44	0.68	0.75	2	12
	Total	3.67	6.18	5.87	4.57	8.73	4.11	23	87

The bacterial diversity (Shannon, Simpson index, Evenness and Brillouin) and OTUs richness (Fisher_alpha, Berger-Parker and Chao1) were estimated at 97% sequence similarity from each BPH sample. The female adult BPH (BPH-F6-TN1 and BPH-F16-IR36) samples revealed highest alpha-diversity among the BPH samples. BPH-F0, F0 generation female adult BPH; BPH-F6-TN1, F6 generation female adult BPH isolated from susceptible, TN1, host; BHP-F16-TN1, F16 generation female adult BPH isolated from TN1; BPH-F6-IR36, F6 generation female adult BPH isolated from resistant, IR36, host; BPH-F16-IR36, F16 generation female adult BPH isolated from IR36; BPH-F6-RH, F6 generation female adult BPH isolated from resistant, RH, host; and BPH-F16-RH, F16 generation female adult BPH from RH

**Table 2:** Principal coordinate analysis (PCoA)-based pair-wise comparison of bacterial diversity. Eighty-seven bacterial OTUs sequences of the BPH samples were used to calculate the Bonferroni corrected *P*-value

	BPH-F0	BPH-F6-TN1	BPH-F16-TN1	BPH-F6-IR36	BPH-F16-IR36	BPH-F6-RH	BPH-F16-RH
BPH-F0		1	0.015	1	0.094	0.023	0.000
BPH-F6-TN1	0.739		0.254	1	0.094	1	0.687
BPH-F16-TN1	0.956	0.864		1	0.005	0.065	0.002
BPH-F6-IR36	0.550	0.205	0.487		1	1	1
BPH-F16-IR36	0.909	0.909	0.970	0.392		0.069	0.024
BPH-F6-RH	0.948	0.683	0.922	0.533	0.920		0.011
BPH-F16-RH	0.987	0.794	0.980	0.559	0.947	0.961

**Table 3:** Beta**-**diversity, Whittaker indices, and pair-wise comparisons between the BPH samples based on bacterial OTUs

Sample name	BPH-F0	BPH-F6-TN1	BPH-F16-TN1	BPH-F6-IR36	BPH-F16-IR36	BPH-F6-RH	BPH-F16-RH
BPH-F0	0	0.333	0.430	0.600	0.430	0.333	0.200
BPH-F6-TN1	0.333	0	0.143	0.600	0.143	0.333	0.200
BPH-F16-TN1	0.430	0.143	0	0.666	0.250	0.430	0.333
BPH-F6-IR36	0.600	0.600	0.600	0	0.666	0.600	0.500
BPH-F16-IR36	0.430	0.143	0.250	0.666	0	0.143	0.333
BPH-F6-RH	0.333	0.333	0.430	0.600	0.143	0	0.200
BPH-F16-RH	0.200	0.200	0.333	0.500	0.333	0.200	0

### Distribution of operational taxonomic units (OTUs) and relative abundance analysis

The identified diverse bacterial species in the BPH samples were classified at different taxonomic levels, phyla, classes, orders, families, and genera (see Additional file 2). The diversity of OTUs at the phylum level across all BPH samples was determined as follows: Proteobacteria, Actinobacteria, Bacteroidetes, Firmicutes, Fusobacteria, Euryarchaeota, p_Unclassified, p_ Unclassified and p_Unclassified (see Additional file 2). Gene Cluster 3.0 and TreeView software was used to generate a heat map representation of the OTUs, and the results evidently revealed the bacterial diversity and the relative abundance of OTUs in the bacterial composition (phylum level) among the BPH samples (BPH**-**F0, BPH**-**F6**-**TN1, BPH**-**F16**-**TN1, BPH**-**F6**-**IR36, BPH**-**F16**-**IR36, BPH**-**F6**-**RH, and BPH**-**F16**-**RH) (Fig. 1c).

**Fig. 2:**
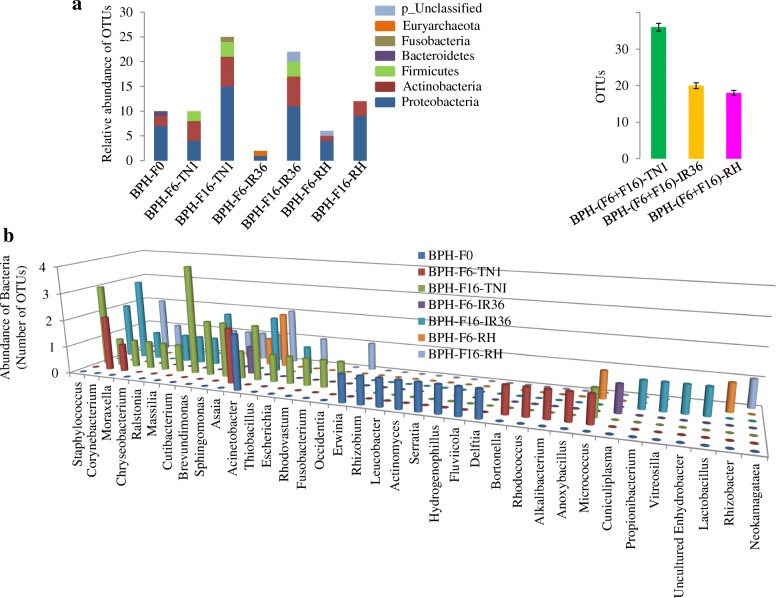
Relative abundance of different bacterial communities among all BPH samples at the level of (**a**) phylum, (**b**) genus, and (**c**) comparative study of the bacterial population in the BPH (at F6 + F16 generations) samples collected from susceptible (TN1) and two resistant (IR36 and RH) rice varieties

With a total of 51 bacterial OTUs, Proteobacteria was observed as the predominant phylum among the BPH samples. Proteobacteria alone shared 7, 4, 15, 1, 11, 4 and 9 of the total OTUs in the samples BPH**-**F0, BPH**-**F6**-**TN1, BPH**-**F16**-**TN1, BPH**-**F6**-**IR36, BPH**-**F16**-**IR36, BPH**-**F6**-**RH and BPH**-**F16**-**RH, respectively (Fig. 2a). However, bacterial OTUs identified as Actinobacteria were the second dominant phylum, and Actinobacteria shared 22 OTUs of the total OTUs classified. The remaining 8, 1, 1, 1 and 3 of the total OTUs represented the phylum Firmicutes, Bacteroidetes, Fusobacteria, Euryarchaeota and p_Unclassified, respectively (Fig. 2a, see Additional file 3). The predominant bacterial OTUs, belonging to Proteobacteria among the BPH samples (Fig. 2b), represented the genera *Acinetobacter* (10 OTUs); *Corynebacterium* (8 OTUs); *Asaia* and *Cutibacterium* (6 OTUs each); *Staphylococcus* and *Brevundimonas* (5 OTUs each); *Moraxella* (4 OTUs); *Micrococcus*, *Sphingomonas* and *Escherichia* (3 OTUs each); and *Ralstonia*, *Massilia* and *Occidentia* (2 OTUs each); *Fusobacterium*, *Rhizobium*, *Erwinia*, *Serratia*, *Leucobacter*, *Actinomyces*, *Fluviicola*, *Rhodococcus*, *Alkalibacterium*, *Anoxybacillus*, *Delftia*, *Hydrogenophilus*, *Bartonella*, *Chryseobacterium*, *Cuniculiplasma*, *Propionibacterium*, *Lactobacillus*, *Rhizobacter*, *Neokamagataea*, *Thiobacillus*, *Rhodovastum*, *Vitreoscilla*, and Uncultured *Enhydrobacter* (1 OTUs each).

### Comparative analysis and identification of bacterial populations between BPH samples collected from susceptible and resistant rice varieties

To study the comparative abundance of the bacterial species across the BPH samples, we organized the different BPH (F6 and F16 generations) samples, isolated from susceptible (TN1) and resistant (IR36 and RH) rice varieties, into three groups: BPH**-**(F6 + F16)**-**TN1, BPH**-(**F6 + F16)**-**IR36 and BPH**-**(F6 + F16)**-**RH samples (Fig. 2c**,** see Additional file 4). We observed that the number of bacterial species was higher in the BPH**-**(F6 + F16)**-**TN1 sample and that the number of bacterial species was lower in the BPH**-**(F6 + F16)**-**RH sample (Fig. 2c**)**. A total of 36, 20 and 18 bacterial species were detected in the BPH**-**(F6 + F16)**-**TN1, BPH**-**(F6 + F16)**-**IR36 and BPH**-**(F6 + F16)**-**RH samples, respectively (see Additional file 4). Comparing the bacterial populations of the BPH**-**(F6 + F16)**-**TN1 sample with the BPH**-**(F6 + F16)**-**IR36 sample, *Micrococcus*, *Staphylococcus*, *Moraxella*, *Chryseobacterium*, *Cutibacterium*, *Sphingomonas*, *Asaia*, *Thiobacillus*, *Rhodovastum*, *Fusobacterium*, *Occidentia*, *Bartonella*, *Rhodococcus*, *Alkalibacterium*, and *Anoxybacillus* were lower in the BPH-(F6 + F16)-IR36 sample (Fig. 2b**,** see Additional file 4), while upon comparison of the bacterial populations in the BPH**-(**F6 + F16)**-**TN1 sample with the BPH**-**(F6 + F16)**-**RH sample, the bacterial population of *Micrococcus*, *Staphylococcus*, *Corynebacterium*, *Moraxella*, *Chryseobacterium*, *Ralstonia*, *Massilia*, *Cutibacterium*, *Brevundimonas*, *Sphingomonas*, *Asaia*, *Thiobacillus*, *Rhodovastum*, *Fusobacterium*, *Occidentia*, *Bartonella*, *Rhodococcus*, *Alkalibacterium*, and *Anoxybacillus* were lower in the BPH**-**(F6 + F16)**-**RH sample (Fig. 2b**,** see Additional file 4). In contrast, *Cuniculiplasma*, *Propionibacterium*, *Vitreoscilla*, uncultured *Enhydrobacter*, and *Lactobacillus* were found to be higher in number in the BPH**-**(F6 + F16)**-**IR36 sample, while *Acinetobacter*, *Rhizobacter*, and *Neokamagataea* were found to be higher in the BPH**-**(F6 + F16)**-**RH sample (Fig. 2b**,** see Additional file 4). These decreases or increases in the number of bacterial species may affect the survival rates of BPHs on resistant (IR36 and RH) rice varieties compared with the susceptible (TN1) rice varieties.

### Specific and shared bacterial population

**Fig. 3:**
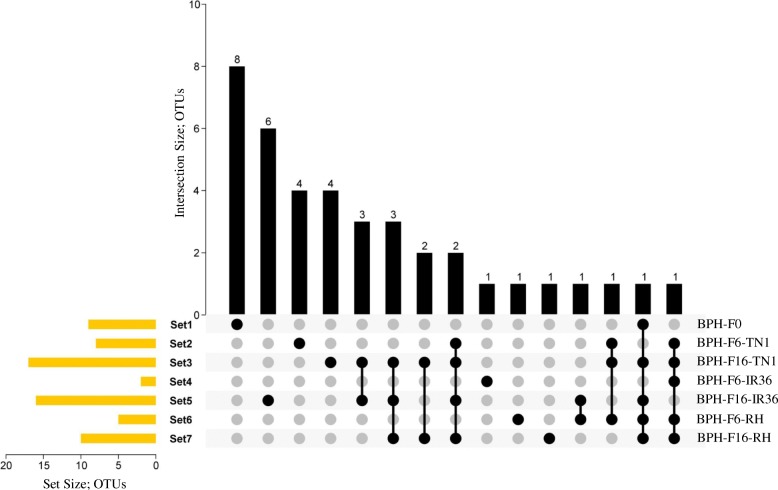
Identification of specific and shared bacterial populations among the BPH samples

The number of specific and shared OTU sequences present in the BPH samples were analysed using a Venn diagram (TBtools (v0.6653) and http://bioinformatics.psb.ugent.be/cgi-bin/liste/Venn/calculate_venn.htpl) (Fig. 3, see Additional file 5). Thirty-nine specific and 14 shared OTU sequences were identified across all samples. *Acinetobacter* and *Asaia* species were shared between five BPH samples, while *Staphylococcus*, *Ralstonia*, *Massilia*, *Sphingomonas*, *Occidentia* and uncultured bacteria were shared between only two BPH samples. The remaining 25 bacterial species were specific for each BPH sample (Fig. 3, see Additional file 5).

### Dynamic variability in the occurrence of *Asaia* in different BPH samples collected from susceptible and resistant rice varieties

**Fig. 4:**
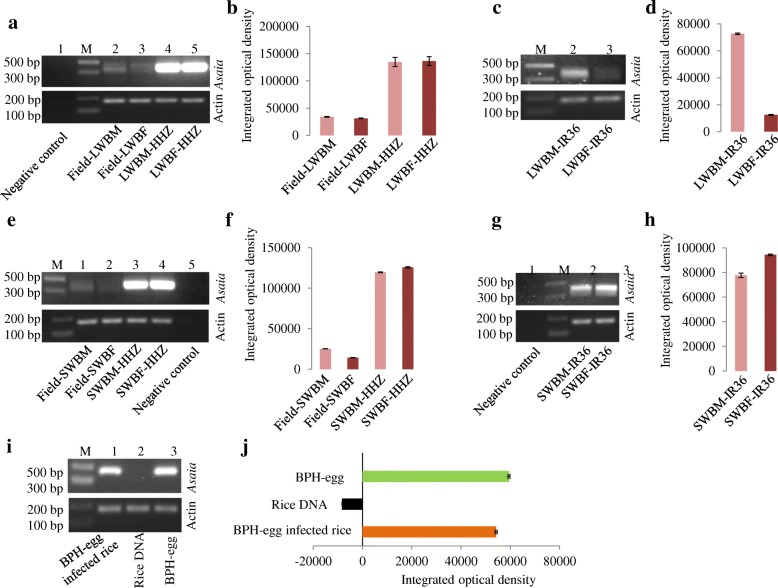
Semiquantitative PCR of *Asaia* and **b**, **d**, **f**, and **h** image analyses of the agarose gel in ‘**a**, **c**, **e**, and **g**’, for quantifying the abundance of *Asaia* in different long- and short-winged adult BPH samples. The actin gene served as the internal control. Field**-**LWBM: field**-**collected long-winged adult male BPH; Field**-**LWBF: field-collected long-winged adult female BPH; LWBM**-**HHR: long-winged adult male BPH collected from the laboratory “HHZ” (susceptible) rice variety; LWBF**-**HHR: long-winged adult female BPH collected from laboratory HHZ; LWBM**-**IR36: long-winged adult male BPH collected from the “IR36” (resistant) rice variety; LWBF**-**IR36: long-winged adult female BPH collected from IR36; Field**-**SWBM: field**-**collected short-winged adult male BPH; Field**-**SWBF: field**-**collected short-winged adult female BPH; SWBM**-**HHR: short-winged adult male BPH collected from the laboratory “HHZ” (susceptible) rice variety; SWBF**-**HHR: short-winged adult female BPH collected from laboratory HHZ; SWBM**-**IR36: short-winged adult male BPH collected from the “IR36” (resistant) rice variety; SWBF**-**IR36: short-winged adult female BPH collected from IR36. ImageJ analyses of the agarose gel in ‘i’, for quantifying abundance of *Asaia* in BPH**-**egg and BPH**-**egg infected rice samples

The RNA-seq results revealed that *Asaia* was the most common bacterial genus between the BPH samples isolated from different rice varieties. *Asaia* have all of the necessary ecological characteristics (discussed in the discussion section), which indicate its potential to be a member of the paratransgenic tool against the BPH. To study the presence of *Asaia* in the different BPH samples, the *Asaia*-specific 16S rRNA gene was amplified by PCR using an *Asaia***-**specific primer (Fig. 4a-h). Amplification of the *Asaia***-**specific 16S rRNA gene (400 bp) was detected in the long-winged adult (male and female) BPH (LWBM**-**HHZ and LWBF**-**HHZ) samples collected from the “HHZ” susceptible rice variety. However, we noticed a weak PCR signal for *Asaia* in the field**-**collected long-winged adult BPH (Field**-**LWBM and Field**-**LWBF) samples (Fig. 4a, b). Furthermore, amplification of the *Asaia***-**specific 16S rRNA gene (400 bp) was observed in the long-winged male BPH (LWBM**-**IR36) sample, but a weak PCR signal for *Asaia* was observed in the long-winged female BPH (LWBF**-**IR36) sample collected from the resistant rice IR36 (Fig. 4c, d). Conversely, amplification of the *Asaia***-**specific 16S rRNA gene (400 bp) was detected in the short-winged adult (male and female) BPH (SWBM**-**HHZ and SWBF**-**HHZ) samples from the “HHZ” susceptible rice (Fig. 4e-h), while we noticed a weak PCR signal for *Asaia* in the field**-**collected short-winged adult (male and female) BPH (Field**-**SWBM and Field**-**SWBF) samples (Fig. 4e, f). Furthermore, amplification of the *Asaia***-**specific 16S rRNA gene (400 bp) was observed in the BPH (SWBM**-**IR36 and SWBF**-**IR36) samples (Fig. 4g, h). Each amplified PCR product, *Asaia***-**specific 16S rRNA gene (400 bp), of the BPH samples was commercially sequenced using the *Asaia***-**specific primers (discussed in the Methods section) at www.igebiotech.com, Guangzhou, China, and the obtained sequencing data were reconfirmed as *Asaia* sp. using the NCBI**-**BLAST 16S rRNA database (data not shown). Nevertheless, there is a need to study the significant role of *Asaia* within their insect host.

### Identification of *Asaia* in the BPH eggs and BPH-egg-infected rice

To recognize the possible role of *Asaia* in the interaction between the BPH and its rice host, we carried out PCR amplification of the *Asaia*-specific 16S rRNA gene (400 bp) from BPH**-**egg, BPH**-**egg**-**infected and uninfected rice tissue (HHZ susceptible rice) samples. Amplification of the *Asaia*-specific gene (400 bp) was detected in the BPH eggs and BPH**-**egg infected rice samples, but there was no amplification of the *Asaia***-**specific gene in the uninfected rice samples (Fig. 4i, j). These PCR products, *Asaia***-**specific 16S rRNA gene (400 bp), were sequenced using the *Asaia***-**specific primers (discussed in the methods section) at www.igebiotech.com, Guangzhou, China, and Sanger sequencing generated sequence data that reconfirmed the sequences as belonging to *Asaia* sp. using the NCBI**-**BLAST 16S rRNA database (data not shown). The detection of the PCR signal of the *Asaia***-**specific gene in BPH**-**egg-infected rice but not in uninfected rice revealed that this bacterium might have an important role in the interaction between the BPH eggs and the host plant upon which they are laid. However, this requires further verification.

## Discussion

The insect-associated bacterial community is one of the key determinants of insect physiology, but there is limited research on the functionality of the BPH bacterial community during the interaction between BPH and rice. The small size of BPH eggs and poor artificial food make research on the BPH difficult [18]. Therefore, this investigation was carried out to identify the bacterial population and to determine its influential role in the interaction between the BPH and rice [19–22]. Further, it would be worth studying whether BPHs feeding on susceptible and resistant rice genotypes can affect survival due to an increase or decrease in the number of favourable bacteria within them.

This project described various bacterial communities in seven different samples of BPHs. Proteobacteria OTUs were the predominant constituents in all BPH samples, possibly because the feeding stage of the BPH on rice possessed Proteobacteria, which could then be relocated from the eggs to adults [23]. Furthermore, the BPH samples, depicting only the feeding stage, revealed distinct alpha-diversity (Table 2). The previous investigation characterized Proteobacteria as the major bacterial community structure in the habitat [24] due to their effective colonization [25, 26]. The Proteobacteria community has also been revealed as the major constituent in the Asian rice gall midge, a monophagous dipteran insect [19], *Drosophila*, a polyphagous [27] dipteran insect and blood feeder bugs i.e., *Cimex lectularius* [28], *Aedes* sp. [29] and ticks [30].

The bacterial microbiomes in the BPHs contained both unique and abundant OTUs. These unique species, which were unique to a respective sample, may be indicative of their important role in BPH physiology and may influence their cooperation with the rice plant. The survival rates of nymphs (BPH) have been reported to be significantly lower on resistant rice hosts than on susceptible rice hosts [31, 32]. Compared to the bacterial community noted in the BPH**-**(F6 + F16)**-**TN1 sample, the BPH**-**(F6 + F16)**-**IR36 and BPH**-**(F6 + F16)**-**RH samples from the resistant rice (IR36 and RH) hosts revealed a lower bacterial population from the genera *Micrococcus, Staphylococcus, Corynebacterium, Moraxella, Chryseobacterium, Ralstonia, Massilia, Cutibacterium, Brevundimonas, Sphingomonas, Asaia, Thiobacillus, Rhodovastum, Fusobacterium, Occidentia, Bartonella, Rhodococcus, Alkalibacterium,* and *Anoxybacillus* (see Additional file 4), although there was an enhanced bacterial population from the genera *Cuniculiplasma*, *Propionibacterium*, *Vitreoscilla*, uncultured *Enhydrobacter*, *Lactobacillus*, *Acinetobacter*, *Rhizobacter*, and *Neokamagataea* (see Additional file 4)*.* This decrease in various bacterial species in the BPH**-**(F6 + F16)**-**IR36 and BPH**-**(F6 + F16)**-**RH samples may be the cause for BPH**-**(F6 + F16)**-**IR36 and BPH**-**(F6 + F16)**-**RH mortality because host invariability and demise are equated to change in the native bacterial population, as hypothesized by researchers [33].

*Acinetobacter, Corynebacterium* and *Asaia* are opportunistic bacteria that cause infections in humans [34–36]. These 3 bacterial species were identified in all BPH samples. The ten observed *Acinetobacter* in the samples had maximum sequence identity to *A. vivianii*, *A. radioresistens*, *A. halotolerans*, *A. soli*, *A johnsonii* and *A. calcoaceticus* (see Additional file 2). Furthermore, it has been reported that plasmids are key elements in prokaryotic evolution and their adaptation to variable environmental circumstances [37]. The existence and broad diversity of plasmids in *Acinetobacter* are well known for natural transformation [38], metabolic activities or metal resistance [39–41] in their host. However, in the context of BPH, there is still no report regarding whether a similar function of *Acinetobacter* plasmids within the BPH shows any impact on insight into the BPH**-**microbe interaction. This is yet to be proven.

Insect cells cannot secrete all kinds of enzymes and thus require microbes to supply specific enzymes for nutrient metabolism [42]. A recent study observed that enzymes are secreted by microbes into their insect host to balance the insect life cycle [43]. In our study, *Corynebacterium* and *Staphylococcus* were present among the BPH samples, and we speculate that these microbes may be involved in manipulating the biological functions of their insect host [42, 44, 45]. Nevertheless, this is yet to be elucidated.

The presence of *Asaia* sp. in agricultural pests such as *Nilaparvata lugens* [46], *Scaphoideus titanus* [47], and *Pieris rapae* [48] has previously been demonstrated. A previous study reported that *Asaia* was observed only in front of the ovipositor sheath in the biotype 2 (Mudgo, carrying the resistant gene (*Bph1*) female BPH [46]. In our study, all RNA**-**sequencing data showed that *Asaia* was present in all BPH (excluding BHP**-**F0 and BPH**-**F16**-**IR36) samples isolated from susceptible (TN1) and resistant (IR36 and RH) rice varieties, and further PCR signals reconfirmed the presence of *Asaia* sp. in all BPH samples collected from field, susceptible (Huang Hua Zhan) and resistant (IR36), rice varieties. In the case of *Gluconacetobacter* [46], *Asaia* may contribute to metabolic functions that influence the adaptation of the BPH to both susceptible and resistant rice varieties. However, more information is required to confirm this hypothesis.

Furthermore, *Asaia* have also been reported in multiple symbioses with the mosquito. They influence the interaction with both *Plasmodium* and *Anopheles* [49], possess strong adhesive properties [50], act as an immune**-**modulator activating antimicrobial peptide expression [49], accelerate larval development [51, 52], are transmitted from mother to offspring likely by a mechanism of egg smearing [53], are capable of cross-colonizing insects of phylogenetically distant genera and order [54], are proposed as a tool for the control of mosquito**-**borne diseases, specifically malaria [55, 56], and have positive markers for functional nitrogenise activity [57]. Therefore, a similar role is likely to be possible in the BPH as well. In our study, *Asaia* was observed in BPH**-**egg and BPH**-**egg**-**infected rice stem tissues but was absent in uninfected rice stem tissues. *Asaia* may transfer from female BPHs to rice, likely mediated by an egg-smearing mechanism while BPHs are laying eggs inside the rice stem. The strong adhesive and antibacterial properties of *Asaia* may influence the attachment and survival of the eggs on rice. Taking this observation further, we speculate that *Asaia* may also play a role in the fitness of the BPH and that its cross-colonization property may support its use as a paratransgenic tool for the control of BPH in the infestation of rice. However, this requires further study.

## Conclusions

This study demonstrated that members of Proteobacteria were predominant in all BPH samples. The BPH**-**associated bacterial diversity may be due to the recruitment of various microbiomes from the extrinsic habitat. This diverse bacterial population can play an important role in BPH**-**rice interactions and influence BPH survival in rice (susceptible and resistant) hosts. Furthermore, the thorough *Asaia* documentation thus confirmed and established its interaction with adult BPHs, BPH eggs and rice. Now, with the extensive bacterial population catalogue thus established, this study will facilitate future experiments including the interaction of bacterial species with the insect phenotype. Even more broadly, it is necessary to further investigate the bacterial effect on insect associations with the host and to use native bacteria as a paratransgenic tool for insect control.

## Methods

### Total RNA-sequence analysis for bacterial populations in different BPH samples

Laboratory strains of BPH were collected in September 2007 from Guangdong Academy of Agricultural Sciences (GAAS), Guangzhou, in Guangdong Province, China, and a colony has been reared continuously on susceptible and resistant rice varieties in our laboratory since then. Different generations (F0, F6, and F16) of adult female (each 15) BPH samples from susceptible (TN1) and resistant rice (IR36 carrying the *bph2* resistance gene and RH carrying the *Bph3* resistance gene) [58] varieties were used for the current study. Total RNA was isolated from adult BPH females using a total RNA extraction kit (Omega, Norcross, GA, USA) according to the manufacturer’s instructions. Library construction and sequencing were performed by Vazyme Biotech Co., Ltd. (Nanjing, China) using the Illumina-HiSeq 2500 platform and PE100 as a sequencing strategy (data not shown in this manuscript). A total of 54.61 GB high quality reads (data not shown in this manuscript) were obtained from the BPH samples. An average of 7.8 G reads from seven different BPH samples named BPH**-**F0 (F0 generation), BPH**-**F6**-**TN1 (F6 generation BPH from TN1), BHP**-**F16**-**TN1 (F16 generation BPH from TN1), BPH**-**F6**-**IR36 (F6 generation BPH from IR36), BPH**-**F16**-**IR36 (F16 generation BPH from IR36), BPH**-**F6**-**RH (F6 generation BPH from RH) and BPH**-**F16**-**RH (F16 generation BPH from RH) were analysed to identify the microbial community structure across the samples using the 16S ribosomal RNA Sequences database from National Center for Biotechnology Information (NCBI), USA (https://www.ncbi.nlm.nih.gov) (see Additional files 6, 7, 8, 9, 10, 11 and 12).

### Sample collection

To study the dynamics of *Asaia* in the short- and long-winged (adults; male and female) BPHs, the BPH samples were randomly collected from the entire rice field in Shaoguan city in Guangdong Province, China and stored in 100% alcohol. The rest of the BPH samples used in this study were maintained in the greenhouse at State Key Laboratory of Biocontrol, Sun Yat-sen University, Guangzhou in Guangdong Province, China under standard conditions [59]. The procedure described by Ojha et al. [19] was applied to grow different rice varieties. The BPHs (50 each of females and males) were released on 10**–**15**-**day**-**old plants, and on day 1, the plastic trays with plants were transferred to a humidity (70–90%) chamber for the process of egg incubation. The adult BPHs were collected from “Huang Huas Zhan” (HHZ, a Chinese susceptible rice variety) and IR36. Entomological needles were used to isolate intact eggs from “HHZ” rice within 48 h and preserved in alcohol (100%). Nearly 200 intact eggs were collected and used for the entire experiment. Thirty short- and long-winged BPH adults were also collected from field rice, both susceptible and resistant rice varieties. To study the *Asaia* identification on the rice host, 300 mg of BPH egg**-**infected and uninfected tissues of susceptible rice were collected.

### Isolation of DNA from the BPH and rice samples

The collected BPH samples were frozen at **−** 20 °C for 5 min, immersed in 5% NaOCl (sodium hypochlorite), and rinsed with sterile distilled water five times to remove surface microorganisms. Total DNA was isolated from BPH adults and eggs using the Insect DNA kit (Omega Bio**-**tek, Inc., Norcross, GA, USA) according to the manufacturer’s instructions. Total DNA from infected and uninfected rice tissues was extracted using the Plant DNA Kit (Omega Bio**-**tek, Inc., Norcross, GA, USA) according to the manufacturer’s instructions. DNA concentrations were estimated using a NanoDrop 2000 spectrophotometer (Thermo Fisher Scientific, USA).

### Primer design and PCR amplification of *Asaia* from different BPH and rice samples

The *Asaia***-**specific primers were designed using the *Asaia***-**specific 16S rRNA gene sequences belonging to the accession numbers:**-** NR_122089.1, NR_112880.1, NR_024810.1, NR_114144.1, NR_041564.1, NR_113849.1, NR_024728.1, NR_112879.1, NR_112953.1, NR_113845.1, and NR_024738.1 (see Additional file 13). PCR amplification of the ~ 400 bp 16S rRNA gene sequence of *Asaia* was performed using the *Asaia* primers (forward: 5′**-**GGCGCGTAGGCGGTTTACAC**-**3′ and reverse: 5′**-**TGCGCGTTGCTTCGAATTAAACCA**-**3′) to evaluate their presence in various samples of BPHs and rice. *Actin***-**gene (NCBI**-**GenBank: EU179846.1) specific-primers (forward: 5′**-**TGCGTGACATCAAGGAGA**-**3′ and reverse: 5′**-**TGTTCCAGCCTTCCTT**-**3′) were used and PCR**-**amplified ~ 174 bp actin gene, a housekeeping gene used as an internal control for the normalization of PCR data. Ten ng DNA of short- and long-winged (adults), egg, egg**-**infected and uninfected rice tissues were used as the template for PCR, and the PCR volume was 25 μl. PCRs were performed with 200 μM dNTPs (New England Biolabs Inc.), 0.5 U Q5 Hot start high**-**fidelity *Taq* DNA polymerase (New England Biolabs Inc.), and 13 μM primers (each). The thermal cycler protocol was 98 °C for 5 min, 35 cycles at 98 °C for 30 s, 56 °C for 45 s, and 72 °C for 30 s, and a final 5 min extension at 72 °C. The PCR conditions for actin**-**gene amplification from each BPH sample were 94 °C for 5 min, 35 cycles at 94 °C for 30 s, 54 °C for 30 s, and 72 °C for 45 s, and a final 5 min extension at 72 °C. Amplicons were run on 1% (w/v) agarose gel to analyse band size. The EtBr**-**stained gels were viewed and photographed in our gel documentation system (GL212PRO Imager, Carestream Health Inc., USA), and captured-gel images were saved in JPEG format. Then, ImageJ software (https://imagej.nih.gov/ij/) was used to estimate the relative colour intensities of each amplicons.

### Statistical analysis

The normal distribution and alpha diversity (Shannon and Simpson’s diversity indices, Evenness, Fisher alpha, Brillouin, Fisher alpha, Berger**-**Parker and Chao1) index were used to evaluate the differences in bacterial populations between BPH samples (obtained from both susceptible and resistant rice varieties) used in the current study. PCoA plots were plotted using PAST (Palaeontological Statistics, version 3) software. To show the relative abundance of microbial phyla for the respective BPH samples, heat maps were created using Gene Cluster 3.0 and TreeView software.

## Additional files

Additional file 1: Normal distribution test of bacterial population in the BPH samples. (DOCX 14 kb)

Additional file 2: Details of OTU ID and its NCBI**-**blast, 16S rRNA database, and details of the closest-matched accession numbers for BPH**-**F0, BPH**-**F6**-**TN1, BPH**-**F16**-**TN1, BPH**-**F6**-**IR36, BPH**-**F16**-**IR36, BPH**-**F6**-**RH, and BPH**-**F16**-**RH samples. (PDF 92 kb)

Additional file 3: Overview of the bacterial population across the BPH samples. (PDF 85 kb)

Additional file 4: Comparison of the bacterial populations across the BPH (F6 + F16 generation BPHs collected from susceptible, TN1, and resistant, IR36 and RH, rice varieties) samples at the genera level. Reduction and increase in the bacterial population across the BPH (F6 + F16 generation BPHs collected from the susceptible, TN1, rice variety) samples when compared with the BPH (F6 + F16 generation BPHs collected from resistant, IR36 and RH, rice variety) samples at the genus level. (DOCX 17 kb)

Additional file 5: Identification of a unique bacterial population among the BPH samples. Total number of unique bacteria among the BPH samples (a), shared bacteria between the BPH samples (b), and (c) specific bacterial name among the BPH samples. (DOCX 16 kb)

Additional file 6: Bacterial sequences of the F0 generation BPHs. (PDF 90 kb)

Additional file 7: Bacterial sequences of the F6 generation BPHs from the susceptible, TN1, rice variety. (PDF 84 kb)

Additional file 8: Bacterial sequences of the F16 generation BPHs from the TN1 rice variety. (PDF 86 kb)

Additional file 9: Bacterial sequences of the F6 generation BPHs from the resistant, IR36, rice variety. (TXT 22 kb)

Additional file 10: Bacterial sequences of the F16 generation BPHs from the IR36 rice variety. (XLSX 18 kb)

Additional file 11: Bacterial sequences of the F6 generation BPHs from the resistant, RH, rice variety. (XLSX 10 kb)

Additional file 12: Bacterial sequences of the F16 generation BPHs from the RH rice variety. (PDF 87 kb)

Additional file 13: *Asaia***-**specific primer design. *Asaia***-**specific 16S rRNA gene sequences belonging to the NCBI Accession Numbers NR_122089.1, NR_112880.1, NR_024810.1, NR_114144.1, NR_041564.1, NR_113849.1, NR_024728.1, NR_112879.1, NR_112953.1, NR_113845.1, and NR_024738.1 were used. (PDF 85 kb)

## Data Availability

All data is given in the main body of the manuscript; materials are available from the authors. All sequence data is added to manuscript as additional files (named as Additional files 2, 3, 4, 5, 6, 7, 8, 9, 10, 11 and 12).

## Abbreviations

OTUs: operational taxonomic units
PCR: polymerase chain reaction
RH: Rathu Heenati
TN1: Taichung Native1
