# Supplementary material for: A comparative study of microbial community and dynamics of Asaia in the brown planthopper from susceptible and resistant rice varieties
Source: BMC Microbiol. 2019 Jun 24;19:139. doi: 10.1186/s12866-019-1512-9 (PMC6591912; doi:10.1186/s12866-019-1512-9)
Supplement: Supplementary file 1 — Normal distribution test of bacterial population in the BPH samples. (DOCX 14 kb) [file 12866_2019_1512_MOESM1_ESM.docx]

| Test | BPH-F0 | BPH-F6-TN1 | BPH-F16-TN1 | BPH-F6-IR36 | BPH-F16-IR36 | BPH-F6-RH | BPH-F16-RH |
| --- | --- | --- | --- | --- | --- | --- | --- |
| N | 7 | 7 | 7 | 7 | 7 | 7 | 7 |
| Shapiro-Wilk W | 0.657 | 0.732 | 0.739 | 0.600 | 0.820 | 0.670 | 0.611 |
| p (normal) | 0.001 | 0.008 | 0.009 | 0.0002 | 0.064 | 0.001 | 0.0003 |
| Anderson-Darling A | 1.108 | 0.872 | 0.820 | 1.423 | 0.565 | 1.06 | 1.325 |
| p (normal) | 0.002 | 0.011 | 0.016 | 0.0003 | 0.088 | 0.003 | 0.0006 |

**Table 1|** Normal distribution test of bacterial population in the BPH samples.
