# Supplementary material for: A comparative study of microbial community and dynamics of Asaia in the brown planthopper from susceptible and resistant rice varieties
Source: BMC Microbiol. 2019 Jun 24;19:139. doi: 10.1186/s12866-019-1512-9 (PMC6591912; doi:10.1186/s12866-019-1512-9)
Supplement: Supplementary file 2 — Details of OTU ID and its NCBI-blast, 16S rRNA database, and details of the closest-matched accession numbers for BPH-F0, BPH-F6-TN1, BPH-F16-TN1, BPH-F6-IR36, BPH-F16-IR36, BPH-F6-RH, and BPH-F16-RH samples. (PDF 92 kb) [file 12866_2019_1512_MOESM2_ESM.pdf]

Bacterial sequences of, F16 generation, BPH from TN1 rice variety

>CL615Contig1

GTTGACTTCTGTCGCTGAGATCAACAGCTGTCCCGTTTCAGGCGGGCAGCGACTCTCTGGTCCTACGGGGCCG
TTCAACATCTATGGAGAGTTTGATCCTGGCTCAGGACGAACGCTGGCGGCGTGCTTAACACATGCAAGTCGAA
CGGAAAGGCCCTGCTTTTGTGGGGTGCTCGAGTGGCGAACGGGTGAGTAACACGTGGGTAATCTGCCCTGCA
CTCTGGGATAAGCCTGGGAAACTGGGTCTAATACCGGATAGGAGCGTCCACCGCATGGTGGGTGTTGGAAAG
ATTTATCGGTTTTGGATGGACTCGCGGCCTATCAGCTTGTTGGTGAGGTAATGGCTCACCAAGGCGACGACGG
GTAGCCGGCCTGAGAGGGTGACCGGCCACACTGGGACTGAGAC

>CL2307Contig1

GGAGAGTTTGATCCTGGCTCAGGATGAACGCTGGCGGCGTGCCTAATACATGCAAGTCGAGCGAACAGACGA
GGAGCTTGCTCCTCTGACGTTAGCGGCGGACGGGTGAGTAACACGTGGATAACCTACCTATAAGACTGGGAT
AACTTCGGGAAACCGGAGCTAATACCGGATAACATGTTGAACCGCATGGTTCAACAGTGAAAGACGGTCTTG
CTGTCACTTATAGATGGATCCGCGCCGCATTAGCTAGTTGGTAAGGTAACGGCTTACCAAGGCAACGATGCGT
AGCCGACCTGAGAGGGTGATCGGCCACACTGGAACTGAGACACGG

>CL1408Contig1

CCCGGGAACGTATTCACCGCAGCGTTGCTGATCTGCGATTACTAGCGACTCCGACTTCATGGGGTCGAGTTGC
AGACCCCAATCCGAACTAAGACCGGCTTTCAGAGATTCGCTCAACCTCACAGGCTCGCCACGCGCTGTACCAA
CCATTGTAGCATGCGTGAAGCCCTGGACATAAGGGGCATGATGATTTGACGTCATCCCCACCTTCCTCTCGGCT
TATCACCGGCAGTCCCTCTAGAGTGCCCAACTGAATGCTGGCAACTAGAGGCGAGGGTTGCGCTCGTTGCGG
GACT

>c37148_g1_i1

TAACACATGCAAGTCGAACGATGACTCTCTAGCTTGCTAGAGATGATTAGTGGCGGACGGGTGAGTAACATTT
AGGAATCTGCCTAGTAGTGGGGGATAGCTCGGGGAAACTCGAATTAATACCGCATACGACCTACGGGTGAAA
GGGGGCGCAAGCTCTTGCTATTAGATGAGCCTAAATCAGATTAGCTAGTTGGTGGGGTAAAG

>c38098_g1_i1

GTATCTAATCCTGTTCGCTACCCACGCTTTCGTCCATCAGCGTCAGTTAAATCTTAGTGACCTGCCTTCGCAATT
GGTGTTCTAAGTAATATCTATGCATTTCACCGCTACACTACTTATTCCAGCCACTTCTACTTTACTCAAGACCCG
CAGTATCAATGGCAGTTTCATAGTTAAGCTATGAGATTTCACCACTGACTTACAGGTCCGCCTACGGACCCTTT
AAACCCA

>c39416_g1_i1

GGACTACCAGGGTATCTAATCCTGCTTGCTCCCCACGCTTTCGCACCTCAGCGTCAATACCAGTCCAGTGAGCC
GCCTTCGCCACTGGTGTTCTTCCATATATCTACGCATTCCACCGCTACACATGGAGTTCCACATTCCTCTTCTGT
ACTCAAGTTTCCCAGTTTCCAATGACCCTCCACGGTTAAGCCGTGGGCTTTCACATCAGACTTAAAAAACCGCC
TACGCGCGCTTTACGCCCAATAATTCCGGATAACGCTTGCCACCTACGTATTACCGCGGCTGCTGG

>c41284_g1_i1
GGCTCAGATTGAACGCTGGCGGCATGCCTTACACATGCAAGTCGAACGGCAGCGGGGGTAGCTTGCTACCTG
CCGGCGAGTGGCGAACGGGTGAGTAATACATCGGAACGTGCCCTGTAGTGGGGGATAACTAGTCGAAAGAC

TAGCTAATACCGCATACGACCTGAGGGTGAAAGTGGGGGACCGCAAGGCCTCATGCTATAGGAGCGGCCGA
TGTCTGATTAGCTAGTTGGTGGGGTAAAG

>c41284_g1_i2

GGCTCAGATTGAACGCTGGCGGCATGCCTTACACATGCAAGTCGAACGGCAGCGGGGGTAGCTTGCTACCTG
CCGGCGAGTGGCGAACGGGTGAGTAATACATCGGAACGTACCCAGAAGTGGGGGATAACGCAGCGAAAGTT
GCGCTAATACCGCATACGTTCTACGGAAGAAAGTGGGGGATCTTCGGACCTCATGCTTTTGGAGCGGCC

>c42563_g1_i1

GCCTCATCCTCTCCTTCCTCCGGCTTAACACCGGCGGTCTGTTCAGGGTTCCAAACTCATAGTGGCAACTAAAC
ACGAGGGTTGCGCTCGTTGCGAGACTTAACCCAACACCTTACGGCACGAGCTGACGACAGCCATGCACCACCT
GTGAACCGACCCCAAAAGAGGCACACCCATCTCTGAGCGCTCCCGATCCATGTCAAACCCAGGTAAGGTTCTA
CGCGTTGCATCGAATTAATCCGCATGCTCCGCCGCTTGTGCGGGGCCCCGTCAATTCCTTTGAGTTTTA

>c48559_g1_i1

GTGCCAGCAGCCGCGGTAATACGAAGGGGGCTAGCGTTGCTCGGAATTACTGGGCGTAAAGGGAGCGTAGG
CGGACATTTAAGTCAGGGGTGAAATCCCGGGGCTCAACCTCGGAATTGCCTTTGATACTGGGTGTCTTGAGTA
TGAGAGAGGTGTGTGGAACTCCGAGTGTAGAGGTGAAATTCGTAGATATTCGGAAGAACACCAGTGGCGAA
GGCGACATTCTGGTCTGTTACTGACACTGAGGCTCGAAAGCGTGGGGAGCAAACAGG

>c48559_g1_i2

TTACTGGGCGTAAAGCGCACGTAGGCGGCTTTGTAAGTTAGAGGTGAAAGCCTGGAGCTCAACTCCAGAACT
GCCTTTAAGACTGCATCGCTTGAATCCAGGAGAGGTGAGTGGAATTCCGAGTGTAGAGGTGAAATTCGTAGA
TATTCGGAAGAACACCAGTGGCGAAGGCGACATTCTGGTCTGTTACTGACACTGAGGCTCGAAAGCGTGGGG
AGCAAACAGG

>c48559_g1_i3

GTGCCAGCAGCCGCGGTAATACGAAGGGGGCTAGCGTTGCTCGGAATGACTGGGCGTAAAGGGCGCGTAGG
CGGTTTAGACAGTCAGATGTGAAAATCCGGGGCTCAACCCTGGGACGGCATTTGATACGTTTAGGCTAGAGT
GTGAGAGAGGGTTGTGGAATTCCCAGTGTAGAGGTGAAATTCGTAGATATTCGGAAGAACACCAGTGGCGA
AGGCGACATTCTGGTCTGTTACTGACACTGAGGCTCGAAAGCGTGGGGAGCAAACAGG

>c48667_g1_i1

GGGAGGCAGCAGTGGGGAATATTGGACAATGGGGGGAACCCTGATCCAGCCATGCCGCGTGTGTGAAGAAG
GCCTTTTGGTTGTAAAGCACTTTAAGCGAGGAGGAGGCTACTTAGATTAATACTCTAGGATAGTGGACGTTAC
TCGCAGAATAAGCACCGGCTAACTCTGTGCCAGCAGCCGCGGTAATACAGAGGGTGCAAGCGTTAATCGGAT
TTACTGGGCGTAAAGCGCGCGTAGGCGGCCAATTAAGTCAAATGTGAAATCCCCGAGCTTAACTTGGGAATT
GCATTCGATACTGGTTGGCTAGAGTGTGGGAGAGGATGGTAGAATTCCAGGTGTAGCGGT

>c49837_g1_i1

CGATGATTGCTAGTTGTCGGGATGCATGCATTTCGGTGACGCAGCTAACGCATTAAGCAATCCGCCTGGGGA
GTACGGTCGCAAGATTAAAACTCAAAGGAATTGACGGGGACCCGCACAAGCGGTGGATGATGTGGATTAATT

CGATGCAACGCGAAAAACCTTACCTACCCTTGACATGTCCGGAAGCCTGAAGAGATTTGGGTGTGCCCGAAA
GGGAACTGGAACACAGGTGCTGCATGGCTGTCGTC

>c53721_g1_i1

GAAGAAGGCCTTCGGGTTGTAAAGGACTTTTGTCAGGGAAGAAAAGGATAGGGTTAATACCCCTGTCTGATG
ACGGTACCTGAAGAATAAGCACCGGCTAACTACGTGCCAGCAGCCGCGGTGATACGTAGGGTGCGAGCGTTG
TCCGGATTTATTGGGCGTAAAGGGCTCGTAGGTGGTTGATCGCGTCGGAAGTGTAATCTTGGGGCTTAACCCT
GAGCGTGCTTTCGATACGGGTTGACTTGAGGAAGGTAGGGGAGAATGGAATTCCTGGTGGAGCGGTGGAAT
GCGCAGATATCAGGAGGAACACCAGTGGCGAAGGCGGTTCTCTGGGCCTTTCCTGACGCTGAGGAGCGAAA
GCGTGGGGAGCGAACAGGCTTAGATACCCTGGTAGTCCACGCTGTAAACGGTGGGTACTAGGTGTGGGGTC
CATTCCACGGGTTCCGTGCCGTAGCTAACGCTTTAAGTACCCCGCCTGGGGAGTACGGCCGCA

>c53721_g1_i2

GTGGGGAATATTGCACAATGGGCGAAAGCCTGATGCAGCGACGCCGCGTGAGGGATGACGGCCTTCGGGTT
GTAAACCTCTTTCAGCAGGGACGAAGCGTAAGTGACGGTACCTGCAGAAGAAGCACCGGCTAACTACGTGCC
AGCAGCCGCGGTGATACGTAGGGTGCGAGCGTTGTCCGGATTTATTGGGCGTAAAGGGCTCGTAGGTGGTT
GATCGCGTCGGAAGTGTAATCTTGGGGCTTAACCCTGAGCGTGCTTTCGATACGGGTTGACTTGAGGAAGGT
AGGGGAGAATGGAATTCCTGGTGGAGCGGTGGAATGCGCAGATATCAGGAGGAACACCAGTGGCGAAGGC
GGTTCTCTGGGCCTTTCCTGACGCTGAGGAGCGAAAGCGTGGGGAGCGAACAGGCTTAGATACCCTGGTAGT
CCACGCTGTAAACGGTGGGTACTAGGTGTGGGGTCCATTCCACGGGTTCCGTGCCGTAGCTAACGCTTTAAGT
ACCCCGCCTGGGGAGTACGGCCGCA

>c54875_g1_i1

CGTGAGCACTGCAAAGTACGCTTCTTTAAGGTAAGGAGGTGATCCAACCGCAGGTTCCCCTACGGTTACCTTG
TTACGACTTCACCCCAGTCATGAATCACAAAGTGGTAAGCGCCCTCCCGAAGGTTAAGCTACCTACTTCTTTTG
CAACCCACTCCCATGGTGTGACGGGCGGTGTGTACAAGGCCCGGGAACGTATTCACCGTGGCATTCTGATCCA
CGATTACTAGCGATTCCGACTTCATGGAGTCGAGTTGCAGACTCCAATCCGGACTACGACGCACTTTATGAGG
TCCGCTTGCTCTCGCGAGGTCGCTTCTCTTTGTATGCGCCATTGTAGCACGTGTGTAGCCCTGGTCGTAAGGGC
CATGATGACTTGACGTCATCCCCACCTTCCTCCAGTTTATCACTGGCAGTCTCCTTTGAGTTCCCGGCCGGACC
GCTGGCAACAAAGGATAAGGGTTGCGCTCGTTGCGGGACTTAACCCAACATTTCACAACACGAGCTGACGAC
AGCCATGCAGCACCTGTCTCACGGTTCCCGAAGGCACATTCTCATCTCTGAAAACTTCCGTGGATGTCAAGACC
AGGTAAGGTTCTTCGCGTTGCATCGAATTAAACCACATGCTCCACCGCTTGTGCGGGCCCCCGTCAATTCATTT
GAGTTTTAACCTTGCGGCCGTACTCCCCAGGCGGTCGACTTAACGCGTTAGCTCCGGAAGCCACGCCTCAAGG
GCACAACCTCCAAGTCGACATCGTTTACGGCGTGGACTACCAGGGTATCTAATCCTGTTTGCTCCCCACGCTTT
CGCACCTGAGCGTCAGTCTTCGTCCAGGGGGCCGCCTTCGCCACCGGTATTCCTCCAGATCTCTACGCATTTCA
CCGCTACACCTGGAATTCTACCCCCCTCTACGAGACTCAAGCTTGCCAGTATCAGATGCAGTTCCCAGGTTGAG
CCCGGGGATTTCACATCTGACTTAACAAACCGCCTGCGTGCGCTTTACGCCCAGTAATTCCGATTAACGCTTGC
ACCCTCCGTATTACCGCGGCTGCTGGCACGGAGTTAGCCGGTGCTTCTTCTGCGGGTAACGTCAATGAGCAAA
GGTATTAACTTTACTCCCTTCCTCCCCGCTGAAAGTACTTTACAACCCGAAGGCCTTCTTCATACACGCGGCATG
GCTGCATCAGGCTTGCGCCCATTGTGCAATATTCCCCACTGCTGCCTCCCGTAGGAGTCTGGACCGTGTCTCAG
TTCCAGTGTGGCTGGTCATCCTCTCAGACCAGCTAGGGATCGTCGCCTAGGTGAGCCGTTACCCCACCTACTA
GCTAATCCCATCTGGGCACATCCGATGGCAAGAGGCCCGAAGGTCCCCCTCTTTGGTCTTGCGACGTTATGCG
GTATTAGCTACCGTTTCCAGTAGTTATCCCCCTCCATCAGGCAGTTTCCCAGACATTACTCACCCGTCCGCCACT
CGTCAGCAAAGAAGCAAGCTTCTTCCTGTTACCGTTCGACTTGCATGTGTTAGGCCTGCCGCCAGCGTTCAATC

TGAGCCATGATCAAACTCTTCAATTTAAAAGTTTGATGCTCAAAGAATTAAACTTCGTAATGAATTACGTGTTC
ACTCTTGAGACTTGGTATTCATTTTTCGTCTTGCGACGTTAAGAATCCGTATCTTCGAGTGCCCACACAGATTGT
CTGATAAATTGTTAAAGAGCAGTGCCGCTTTGCTTTTTCTCAGCGGCGCGGG

>c56390_g1_i2

GGTGTGACGGGCGGTGTGTACAAGACCCGGGAACGTATTCACCGTAGCATGCTGATCTACGATTACTAGCGA
TTCCAGCTTCATGTAGTCGAGTTGCAGACTACAATCCGAACTGAGAACAACTTTATGGGATTTGCTTGACCTCG
CGGTTTCGCTGCCCTTTGTATTGTCCATTGTAGCACGTGTGTAGCCCAAATCATAAGGGGCATGATGATTTGAC
GTCATCCCCACCTTCCTCTCGGCTTATCACCGGCAGTCCCTCTAGAGTGCCCAACTGAATGCTGGCAACTAGAG
GCGAGGGTTGCGCTCGTTGCGGGACT

>c57055_g1_i3

GTTGACTTCTGTCGCTGAGATCAACAGCTGTCCCGTTTCAGGCGGGCAGCGACTCTCTGGTCCTACGGGGCCG
TTCAACATCTATGGAGAGTTTGATCCTGGCTCAGGACGAACGCTGGCGGCGTGCTTAACACATGCAAGTCGAA
CGGAAAGGCCCTGCTTTTGTGGGGTGCTCGAGTGGCGAACGGGTGAGTAACACGTGAGTAACCTGCCCTTGA
CTTTGGGATAACTTCAGGAAACTGGGGCTAATACCGGATAGGAGCTCCTGCTGCATGGTGGGGGTTGGAAAG
TTTCGGCGGTTGGGGATGGACTCGCGGCTTATCAGCTTGTTGGTGGGGTAGTGGCTTACCAAGGCTTTGACG
GGTAGCCGGCCTGAGAGGGTGACCGGCCACATTGGGACTGAGAT

>c57055_g2_i1

GGAAGGGTAGCTTGCTACCTGACCTAGCGGCGGACGGGTGAGTAATGCTTAGGAATCTGCCTATTAGTGGGG
GACAACATCTCGAAAGGGATGCTAATACCGCATACGTCCTACGGGAGAAAGCAGGGGATCACTTGTGACCTT
GCGCTAATAGATGAGCCTAAGTCGGATTAGCTAGTTGGTGGGGTAAAGGCCTACCAAGGCGACGATCTGTAG
CGGGTCTGAGAGGATGATCCGCCACACTGGGACTGAGACACGGCCCAGACTCCTACGGGAGGCAGCAG

>c57055_g3_i1

GGCCTAACACATGCAAGTCGAACGAACTCTTCGGAGTTAGTGGCGGACGGGTGAGTAACACGTGGGAACGT
GCCTTTAGGTTCGGAATAACTCAGGGAAACTTGTGCTAATACCGAATGTGCCCTTCGGGGGAAAGATTTATCG
CCTTTAGAGCGGCCCGCGTCTGATTAGCTAGTTGGTGAGGTAAAGGCTCACCAAGGCGACGATCAGTAGCTG
GTCTGAGAGGATGATCAGCCACATTGGGACTGAGACACGGCCCAAACTCCTACGGGAGGCAGCAGTGG

>c57055_g3_i2

GGCCTAACACATGCAAGTCGAACGAAGCCTTCGGGCTTAGTGGCGCACGGGTGCGTAACGCGTGGGAATCTG
CCCCTCGGTTCGGAATAACAGTTAGAAATGACTGCTAATACCGGATGATGACGTAAGTCCAAAGATTTATCGC
CGAGGGATGAGCCCGCGTAGGATTAGCTAGTTGGTGTGGTAAGAGCGCACCAAGGCGACGATCCTTAGCTG
GTCTGAGAGGATGATCAGCCACATTGGGACTGAGACACGGCCCAAACTCCTACGGGAGGCAGCAGTGG

>c57055_g4_i1

TCCTGGCTCAGAGTGAACGCTGGCGGCATGCTTAACACATGCAAGTCGCACGGGCAGCAATGTCAGTGGCGG
ACGGGTGAGTAACGCGTAGGGATTTATCCATAGGTGGGGGATAACACTGGGAAACTGGTGCTAATACCGCAT
GACACCTGAGGGTCAAAGGCGCGAGTCGCCTATGGAGGAGCCTGCGTTCGATTAGCTAGTTGGTTAGGTAAA
AGCTGACCAAGGCGATGATCGATAGCTGGTCTGAGAGGATGATCAGCCACATTGGGACTGAGACACGGCCCA
AACTCCTACGGGAGGCAGCAGTGG

>c77484_g1_i1

AGCGAATGCTAATACCTGATATTATGATTTTAGGGCATCCTAGAATTATGAAAGCTATATGCGCTGTGAGAGA
GCTTTGCGTCCCATTAGCTAGTTGGAGAGGTAACGGCTCACCAAGGCGATGATGGGTAGCCGGCCTGAGAGG
GTGAACGGCCACAAGGGGACTGAGACACGGCCCTTACTCCTACGGGAGGCAGCAGTGG

>c85382_g1_i1

GTATAGATTTCAGATCCTCGGCTTGGTAAGCCATTACCTTACCAACTACCTAATCTTTTTTATAGGCTCTATTTTA
TGGCGGGTTTCCCCTTATTTATTCCTTTTCCATATATTAGTTTATATTCCTATATAAAACTCACCCGTATGCTATG
ATCTAACGATAGTCGTTAACTACGTTCTTTTCATACAACTTGCATGTGTAATGCCGATAGTTAGCGTAAATTCGT
AGCCA
