# Supplementary material for: A comparative study of microbial community and dynamics of Asaia in the brown planthopper from susceptible and resistant rice varieties
Source: BMC Microbiol. 2019 Jun 24;19:139. doi: 10.1186/s12866-019-1512-9 (PMC6591912; doi:10.1186/s12866-019-1512-9)
Supplement: Supplementary file 3 — Overview of the bacterial population across the BPH samples. (PDF 85 kb) [file 12866_2019_1512_MOESM3_ESM.pdf]

Bacterial sequences of, F6 generation, BPH from resistant, IR36, rice variety

>c8525_g1_i1

GTGATCCAGCCGCAGGTTCCCCTACGGCTACCTTGTTTCGACTTCACCCCAGTCGCTGACCCGACCGTGGTCGG
CTGCGTCCTCGCGGTTCGCTCACCGGCTTCGGGTCAAACCAACTCCCATGGTGTGACGGGCGGTGTGTACAAG
GCCCGGGAACGTATTCACCGCGGCATGCTGATCCGCGATTACTAGCGATTCCACCTTCATGCACTCGAGTTGC
AGAGTACAATCCGAACTGAGACGGCTTTTAGAGATCAGCACGATGTCACCATCTAGCTTCCCACTGTCACCGC
CATTGTAGCACGTGTGTAGCCCAGGTCATAAGGGCCATGAGGACTTGACGTCATCCCCACCTTCCTCCGGCTT
GTCACCGGCAGTTCCTCTAGAGTGCCCACCCAAACATGCTGGCAACTAAAGGCAAGGGTTGCGCTCGTTGCG
GGACTTAACCCAACATCTCACGACACGAGCTGACGACAGCCATGCAGCACCTGTGCTGGAGGTCCCTTGCAG
GAAATATCCATCTCTGAATACAGCCTCCACATGTCAAGACCTGGTAAGGTTCTGCGCGTTGCTTCGAATTAAAC
CACATGCTCCACCGCTTGTGCGGGCCCCCGTCAATTCCTTTGAGTTTCAACCTTGCGGCCGTACTCCCCAGGCG
GTGTGCTTAGCGCGTTAGCTTCGACACTGAAAAACTAAGTTCTCCAACATCCAGCACACATCGTTTACAGCGTG
GACTACCAGGGTATCTAATCCTGTTTGCTCCCCACGCTTTCGCGCCTCAGCGTCAGTAGTGAGCCAGGTTGCCG
CCTTCGCCACCGGTGTTCTTCCCAATATCTACGAATTTCACCTCTACACTGGGAATTCCACAACCCTCTCTCACA
CTCTAGCCTAAACGTATCAAATGCCGTCCCAGGGTTGAGCCCCGGATTTTCACATCTGACTGTCTAAACCGCCT
ACGCGCCCTTTACGCCCAGTCATTCCGAGCAACGCTAGCCCCCTTCGTATTACCGCGGCTGCTGGCACGAAGTT
AGCCGGGGCTTCTTCTACAGGTACCGTCATCATCGTCCCCGTCGAAAGTGCTTTACAATCCGAAGACCTTCTTC
ACACACGCGGCATTGCTGGATCAGGCTTGCGCCCATTGTCCAATATTCCCCACTGCTGCCTCCCGTAGGAGTCT
GGGCCGTGTCTCAGTCCCAGTGTGGCTGATCATCCTCTCAGACCAGCTATCGATCATCGCCTTGGTCAGCTTTT
ACCTAACCAACTAGCTAATCGAACGCAGGCTCCTCCATAGGCGACTCGCGCCTTTGACCCTCAGGTGTCATGC
GGTATTAGCACCAGTTTCCCAGTGTTATCCCCCACCTATGGATAAATCCCTACGCGTTACTCACCCGTCCGCCAC
TCACCCCGAAAGGTCCGTGCGACTTGCATGTGTTAAGCATGCCGCCAGCGTTCGCTCTGAGCCAGGATCAAAC
TCTCAGGTTTAACCCTAGCAGTATCAAACTGCTAACATTAACAGCTCAATATAAAACAAAAAAACCGACGTCGT
AGTTTATTAAAAAATACGCCAACAGCTCCTTAGCCTATATCAAACAGACTAATAAAACGCCGCCAACATATCCC
TTCTTATCACAATATTAAATTGTCAATGACCAAA

>CL128Contig1

GGGGGGGGGGGGGGGGGGGGGGGGAAAGGGGGGGGGGTCACGGGCGTGGCCCCGCCCGTGCCCCCCCCT
GCCCCCGTGCCACCCGCCGAGGCAACGTCCTCGCGGGGGGCCCCTGCGAGGGGAGGGTTCACTGGGGGGGT
TCGGGAGTCGGATGACTCGGTAATGATCCCTCCGCTGGTTCACCAACGGAGACCTTGTTACGACTTTTACTTCC
TCTAAATGACCGAGTTCGGAGAGCTTTCCGGCCCTGGGTAGCGGTTGCCCACCCCCCCGGGCCAGTCCGGGC
GCCTCACTGAGCCATTCAATCGGTAGTAGCGACGGGCGGTGTGTACAAAGGGCAGGGACGTAATCAACGCAA
GCTGATGACTTGCGCTTACTAGGGATTCCTCGTTGAAGAGCAATAATTGCAATGCTCTATCCCCAGCACGACG
GAGTTTAACAAGATTACCCGGGCCTTCCGGCCAAGGGAGTTACTCGCTGGCTCCGTCAGTGTAGCGCGCGTGC
GGCCCAGAACATCTAAGGGCATCACAGACCTGTTATTGCCTCAAACTTCCATCGGCTTGAGCCGATAGTCCCTC
TAAGAAGCCGGCGCGCCGCCGCAGCGACGCGGGCTATTTAGCAGGTTAAGGTCTCGTTCGTTATCGCAATTA
AGCAGACAAATCACTCCACCAACTAAGAACGGCCATGCACCACCACCCACAAAATCAAGAAAGAGCTCTCAAT
CTGTCAATCCTCATTGTGTCTGGACCTGGTGAGTTTCCCCGTGTTGAGTCAAATTAAGCCGCAGGCTCCACCCC
TGGTGGTGCCCTTCCGTCAATTTCTTTAAGTTTCAGCCTTGCGACCATACTCCCCCTGGAGCCCAAGCACTTTGA
TTTCTCGTAAGGTGCCGAACGAGTCAAAAAATAACATCGTCCGATCCCTAGTCGGCATAGTTTATGGTTAAGA
CTACGACGGTATCTGATCGTCTTCGATCCCCTAACTTTCGTTCCTGATTAATGAAAACATCCTTGGCAAATGCTT
TCGCAGTAGTGAGTCTTCAATCGATCCAAGAATTTCACCTCTGACGATCGAATACTGATGCCCCCGACTGTCCC
TATTAATCATTACGGCGGTCCTAGAAACCAACAAAATAGAACCGCGCGTCCTATTTCATTATTCCATGCTAATG

TATTCGAAGCATAGGCCTGCCTGGAGCACTCTAATTTTTTCAAAGTAAAAAGTCCTGCTTCCCCCGCCACGCCC
AGTGAAGGGCATGGGGTTCCACAGAGGGAGGGGCCCGGGCCGGGCCAGTACACGCGGTGAGGCGGACCGG
CCAGCCAGGCCCGAGGTTCAACTACGAGCTTTTTAACCACAACAACTTTAATATACGCTATTGGAGCTGGAATT
ACCGCGGCTGCTGGCACCAGACTTGCCCTCCAATTGTTCCTCGTTAAGGGATTTAAATTGTACTCATTCCAATT
ACAAGACCCGAAAGGGCCCTGTATCAGTATTTATTGTCACTACCTCCCCGAGTCGGGATTGGGTAATTTGCGC
GCCTGCTGCCTTCCTTGGATGTAGTAGCCGTTTCTCAGGCTCCTTCTCCGGGGTCGAGCCCTAACCCTCCGTTA
CCCGTTGCAACCATGTTTGGCCACTACCCAAACATCGAAAGTTGATAGGGAAGAAATTTGAATGAACCATCGC
CGGCACAAGGCCGTGCGATCCGCGAAGTTATCATGAATCACCAGTGAGCCCCAGAGGGCATTGGTTTTTGAA
TCTAATAAATACATCCCTTCCGGAGTTGGGATTTTCAGCATGTATTAGCTCTAGAATTACCACGGTTATCCAAG
TAGTAAGGTGCTATCAAATAAACGATAACTTGTGTAATGAGCCATTCGCAGTTTCGCCGTATAATTCGCTTATA
CTTAGACATGCATGGCTTAATCTTTGAGACAAGCATATGACTACTAGCAATCAATGTTGAGTTTGAAGTGAAT
GATAGCTCATGTACTGAAAACAGCCTGTGGATGAACAAATGTATTGCATGAGCTGACAAAATAGTAGGCCTAC
CATTGAACTCAACTCCTAGATATTAGCAAGGAGAGACTTGGCGTTGTTGCCATGACGTTTACGTGTGCGATGA
GGCTGTTCTTCCAGGTGGCAATTTTTTAGCAGAGGATATGTCGACATGAGCTATTAGCTAGTGGATGGATGAT
TGTATGAGATGATCAAACGCAAGGTAGGTAGATAAAACGTCCATACCTACAGCGGGATTCGAACCCACGACA
AGGTAGCACTAGCTACTGAGGGGGTGCAAAGCCTTGTCGCCTGCTCAGGTGGTAATCATATGTGGTGAGGGT
AGCGGGGGTTATTCTTCAGCAGAGTGATACCCCATATGCGTAGTCAGGTTGTAGTACTTGTAGTCAACTTCTTA
TCACACACATCACATGTATATACACTTGCTTAACAACTTTCTTATCCTCATACAACCATCAGGACCTGCGTAAAA
GACTTAATTTAACACCGTGCTATTCAAATAACCCAGGTCTGGGTGAAAGGCCTCGCTTGAAACCGTGTTGTTCA
AATCGCCGCTGGTCGATTGGGAAGAACTTGGGGCCTCTTCATCATCGATTGGCGTTTTTGAGCTGAACGATAA
TTAGCGGAACTCCA
