# Supplementary material for: A comparative study of microbial community and dynamics of Asaia in the brown planthopper from susceptible and resistant rice varieties
Source: BMC Microbiol. 2019 Jun 24;19:139. doi: 10.1186/s12866-019-1512-9 (PMC6591912; doi:10.1186/s12866-019-1512-9)
Supplement: Supplementary file 4 — Comparison of the bacterial populations across the BPH (F6 + F16 generation BPHs collected from susceptible, TN1, and resistant, IR36 and RH, rice varieties) samples at the genera level. Reduction and increase in the bacterial population across the BPH (F6 + F16 generation BPHs collected from the susceptible, TN1, rice variety) samples when compared with the BPH (F6 + F16 generation BPHs collected from resistant, IR36 and RH, rice variety) samples at the genus level. (DOCX 17 kb) [file 12866_2019_1512_MOESM4_ESM.docx]

| S. No. | Bacteria | BPH-(F6+F16)-TN1 | BPH-(F6+F16)-IR36 | BPH-(F6+F16)-RH |
| --- | --- | --- | --- | --- |
| 1 | *Micrococcus* | 2 | 0 | 1 |
| 2 | *Staphylococcus* | 3 | 2 | 0 |
| 3 | *Corynebacterium* | 3 | 3 | 2 |
| 4 | *Moraxella* | 2 | 1 | 1 |
| 5 | *Chryseobacterium* | 1 | 0 | 0 |
| 6 | *Ralstonia* | 1 | 1 | 0 |
| 7 | *Massilia* | 1 | 1 | 0 |
| 8 | *Cutibacterium* | 4 | 1 | 1 |
| 9 | *Brevundimonas* | 2 | 2 | 1 |
| 10 | *Sphingomonas* | 2 | 0 | 1 |
| 11 | *Asaia* | 3 | 1 | 2 |
| 12 | *Acinetobacter* | 2 | 2 | 4 |
| 13 | *Thiobacillus* | 1 | 0 | 0 |
| 14 | *Escherichia* | 1 | 1 | 1 |
| 15 | *Rhodovastum* | 1 | 0 | 0 |
| 16 | *Fusobacterium* | 1 | 0 | 0 |
| 17 | *Occidentia* | 1 | 0 | 1 |
| 18 | *Bartonella* | 1 | 0 | 0 |
| 19 | *Rhodococcus* | 1 | 0 | 0 |
| 20 | *Alkalibacterium* | 1 | 0 | 0 |
| 21 | *Anoxybacillus* | 1 | 0 | 0 |
| 22 | *Micrococcus* | 1 | 0 | 1 |
| 32 | *Cuniculiplasma* | 0 | 1 | 0 |
| 24 | *Propionibacterium* | 0 | 1 | 0 |
| 25 | *Vitreoscilla* | 0 | 1 | 0 |
| 26 | *Uncultured Enhydrobacter* | 0 | 1 | 0 |
| 27 | *Lactobaciilus* | 0 | 1 | 0 |
| 28 | *Rhizobacter* | 0 | 0 | 1 |
| 29 | *Neokamagataea* | 0 | 0 | 1 |
| Total | | 36 | 20 | 18 |

**Table 2**| Comparison of the bacterial populations in across the BPH (F6+F16 generations BPH collected from susceptible, TN1, and resistant, IR36 and RH, rice variety) samples at the genera level. Reduction and increase of bacterial population across the BPH (F6+F16 generations BPH collected from resistant, IR36 and RH, rice variety) samples when compared with the BPH (F6+F16 generations BPH collected from susceptible, TN1, rice variety) samples at the genus level.
