# Supplementary material for: A comparative study of microbial community and dynamics of Asaia in the brown planthopper from susceptible and resistant rice varieties
Source: BMC Microbiol. 2019 Jun 24;19:139. doi: 10.1186/s12866-019-1512-9 (PMC6591912; doi:10.1186/s12866-019-1512-9)
Supplement: Supplementary file 5 — Identification of a unique bacterial population among the BPH samples. Total number of unique bacteria among the BPH samples (a), shared bacteria between the BPH samples (b), and (c) specific bacterial name among the BPH samples. (DOCX 16 kb) [file 12866_2019_1512_MOESM5_ESM.docx]

**Table 3**| Identification of unique bacterial population among the BPH samples. Total number of unique bacteria among the BPH samples (a), shared bacteria between the BPH samples (b), and (c) specific bacterial name among the BPH samples.

a.

| S. No. | Samples name | Number of microbes | Number of Unique microbes |
| --- | --- | --- | --- |
| 1 | BPH-F0 | 10 | 9 |
| 2 | BPH-F6-TN1 | 10 | 8 |
| 3 | BPH-F16-TN1 | 25 | 17 |
| 4 | BPH-F6-IR36 | 2 | 2 |
| 5 | BPH-F16-IR36 | 22 | 16 |
| 6 | BPH-F6-RH | 6 | 5 |
| 7 | BPH-F16-RH | 12 | 10 |
| Overall number of unique microbes | | | 39 |

| S. No. | Samples name | Total | Shared Microbes |
| --- | --- | --- | --- |
| 1 | BPH-F0,BPH-F16-TN1, BPH-F16-IR36,BPH-F6-RH, BPH-F16-RH | 1 | *Acinetobacter* |
| 2 | BPH-F6-TN1, BPH-F16-TN1,BPH-F6-IR36,BPH-F6-RH, BPH-F16-RH | 1 | *Asaia* |
| 3 | BPH-F6-TN1,BPH-F16-TN1, BPH-F16-IR36, BPH-F16-RH | 2 | *Moraxella, Corynebacterium* |
| 4 | BPH-F16-TN1, BPH-F16-IR36, BPH-F16-RH | 3 | *Brevundimonas, Escherichia, Cutibacterium* |
| 5 | BPH-F6-TN1, BPH-F16-TN, 1BPH-F6-RH | 1 | *Micrococcus* |
| 6 | BPH-F16-TN1, BPH-F16-IR36 | 3 | *Staphylococcus, Ralstonia, Massilia* |
| 7 | BPH-F16-TN1, BPH-F16-RH | 2 | *Sphingomonas, Occidentia* |
| 8 | BPH-F16-IR36, BPH-F6-RH | 1 | *Uncultured bacteria* |

b.

c.

| S. No. | Samples name | Total | Unique Microbes |
| --- | --- | --- | --- |
| 1 | BPH-F0 | 8 | *Hydrogenophilus, Rhizobium, Leucobacter, Actinomuces, Fluviicola, Erwinia, Serratia, Delftia* |
| 2 | BPH-F6-TN1 | 4 | *Rhodococcus, Alkalibacterium, Anoxybacillus, Bortonella* |
| 3 | BPH-F16-TN1 | 4 | *Thiobacillus, Chryseobacterium, Rhodovastum, Fusobacterium* |
| 4 | BPH-F6-IR36 | 1 | *Cuniculiplasma* |
| 5 | BPH-F16-IR36 | 6 | *Propionibacterium,* Unclassified *bacterium, Vitreoscilla, Lactobacillus, Uncultured Enhydrobacter, Microbacterium* |
| 6 | BPH-F6-RH | 1 | *Rhizobacter* |
| 7 | BPH-F16-RH | 1 | *Neokomagataea* |
