# Supplementary material for: A comparative study of microbial community and dynamics of Asaia in the brown planthopper from susceptible and resistant rice varieties
Source: BMC Microbiol. 2019 Jun 24;19:139. doi: 10.1186/s12866-019-1512-9 (PMC6591912; doi:10.1186/s12866-019-1512-9)
Supplement: Supplementary file 6 — Bacterial sequences of the F0 generation BPHs. (PDF 90 kb) [file 12866_2019_1512_MOESM6_ESM.pdf]

Bacterial sequences of, F16 generation, BPH from IR36 rice variety

>c17231_g1_i1

ATTAAATTGTTGGTTCAAAACAAGTGTTAGAGATAACACAAATTAGTATTTTATGAGCTAATCAAACATCATAT
TTTTATGGAGAGTTTGATCCTGGCTCAGGATGAACGCTGGCGGCGTGCCTAATACATGCAAGTCGAGCGAAC
AGATAAGGAGCTTGCTCCTTTGACGTTAGCGGCGGACGGGTGAGTAACACGTAAAGAACCTGCCCTCAGGTC
TGGGATAACCACGAGAAATCGGGGCTAATACCGGATGGGTCATCGGACCGCATGGTCCGA

>c36790_g1_i1

GTGCCAGCAGCCGCGGTAATACGTAGGGTGCGAGCGTTGTCCGGAATTACTGGGCGTAAAGGGCTCGTAGG
TGGTTGATCGCGTCGGAAGTGTAATCTTGGGGCTTAACCCTGAGCGTGCTTTCGATACGGGTTGACTTGAGGA
AGGTAGGGGAGAATGGAATTCCTGGTGGAGCGGTGGAATGCGCAGATATCAGGAGGAACACCGATGGCGA
AGGCAGGTTACTGGGCAGTTACTGACGCTGAGGAGCGAAAGCATGGGTAGCGAACAGGATTAGATACCCTG
GTAG

>c36790_g1_i2

GTGCCAGCAGCCGCGGTAATACGTAGGGTGCGAGCGTTGTCCGGAATTACTGGGCGTAAAGGGCTCGTAGG
TGGTTTGTCGCGTCGTCTGTGAAATTCCGGGGCTTAACTCCGGGCGTGCAGGCGATACGGGCATAACTTGAGT
ACTGTAGGGGTAACTGGAATTCCTGGTGTAGCGGTGGAATGCGCAGATATCAGGAGGAACACCGATGGCGA
AGGCAGGTTACTGGGCAGTTACTGACGCTGAGGAGCGAAAGCATGGGTAGCGAACAGGATTAGATACCCTG
GTAG

>c38159_g1_i1

CAGCAGCCGCGGTAATACGGAGGGAGCTAGCGTTATTCGGAATTACTGGGCGTAAAGCGTGCGCAGGCGGT
TTTGTAAGTCTGTCGTGAAAGCCCCGGGCTCAACCTGGGAATTGCGATGGAGACTGCAAGGCTAGAATCTGG
CAGAGGGGGGTAGAATTCCACGTGTAGCAGTGAAATGCGTAGAGATGTGGAGGAACACCAGTGGCGAAGG
CGACCACCTGGGCTAATACTGACACTGAGGTGCGAAAGCGTGGGGAGC

>c38663_g1_i1

GGCGGACGGGTGAGTAACACGTGGATAACCTACCTATAAGACTGGGATAACTTCGGGAAACCGGAGCTAATA
CCGGATAATATTTCGAACCGCATGGTTCGATAGTGAAAGATGGTTTTGCTATCACTTATAGATGGACCCGCGC
CGTATTAGCTAGTTGGTAAGGTAACGGCTTACCAAGGCAACGATACGTAGCCGACCTGAGAGGGTGATCGGC
CACACTGGAACTGAGACACGG

>c38933_g1_i1

GGGAGGCAGCAGTGGGGAATATTGGACAATGGGGGCAACCCTGATCCAGCCATGCCGCGTGTGTGAAGAAG
GCCTTATGGTTGTAAAGCACTTTAAGCGAGGAGGAGGCTCTTTTGGTTAATACCCAAGATGAGTGGACGTTAC
TCGCAGAATAAGCACCGGCTAACTCTGTGCCAGCAGCCGCGGTAATACAGAGGGTGCAAGCGTTAATCGGAT
TTACTGGGCGTAAAGCGCGCGTAGGCGGCCAATTAAGTCAAATGTGAAATCCCCGAGCTTAACTTGGGAATT
GCATTCGATACTGGTTGGCTAGAGTGTGGGAGAGGATGGTAGAATTCCAGGTGTAGCGGT

>c38933_g1_i2
GGGAGGCAGCAGTGGGGAATATTGGACAATGGGGGCAACCCTGATCCAGCCATGCCGCGTGTGTGAAGAAG
GCCTTTTGGTTGTAAAGCACTTTAAGCAGGGAGGAGAGGCTAATGGTTAATACCCATTAGATTAGACGTTACC

TGCAGAATAAGCACCGGCTAACTCTGTGCCAGCAGCCGCGGTAATACAGAGGGTGCGAGCGTTAATCGGAAT
TACTGGGC

>c43855_g1_i1

CGCAAGTAGAGAGGAAGGTGGGGATGACGTCAAATCATCATGCCCCTTATGTCCAGGGCTTCACACATGCTA
CAATGGTCGGTACAACGCGCAGCGACACTGTGAGGTGGAGCGAATCGCTGAAAGCCGGCCTTAGTTCGGATT
GGGGTCTGCAACTCGACCCCATGAAGTCGGAGTCGCTAGTAATCGCAGATCAGCAATGCTGCGGTGAATACG
TTCCCG

>c43855_g1_i2

CGCAAGTAGAGAGGAAGGTGGGGATGACGTCAAATCATCATGCCCCTTATGTCTTGGGCTTCACGCATGCTAC
AATGGCCGGTACAAAGGGCTGCAATACCGTGAGGTGGAGCGAATCCCAAAAAGCCGGTCCCAGTTCGGATTG
AGGTCTGCAACTCGACCTCATGAAGTCGGAGTCGCTAGTAATCGCAGATCAGCAACGCTGCGGTGAATACGTT
CCCGGGCCTT

>c44616_g1_i1

GGGGGCTAGCGTTGCTCGGAATTACTGGGCGTAAAGGGAGCGTAGGCGGACATTTAAGTCAGGGGTGAAAT
CCCGGGGCTCAACCTCGGAATTGCCTTTGATACTGGGTGTCTTGAGTATGAGAGAGGTGTGTGGAACTCCGA
GTGTAGAGGTGAAATTCGTAGATATTCGGAAGAACACCAGTGGCGAAGGCGACACACTGGCTCATTACTGAC
GCTGAGGCTCGAAAGCGTGGGGAGCAAACAGGA

>c45279_g1_i1

CGGAGGTGATCCAACCGCAGGTTCCCCTACGGTTACCTTGTTACGACTTCACCCCAGTCATGAATCACAAAGT
GGTAAGCGCCCTCCCGAAGGTTAAGCTACCTACTTCTTTTGCAACCCACTCCCATGGTGTGACGGGCGGTGTG
TACAAGGCCCGGGAACGTATTCACCGTGGCATTCTGATCCACGATTACTAGCGATTCCGACTTCATGGAGTCG
AGTTGCAGACTCCAATCCGGACTACGACGCACTTTATGAGGTCCGCTTGCTCTCGCGAGGTCGCTTCTCTTTGT
ATGCGCCATTGTAGCACGTGTGTAGCCCTGGTCGTAAGGGCCATGATGACTTGACGTCATCCCCACCTTCCTCC
AGTTTATCACTGGCAGTCTCCTTTGAGTTCCCGGCCGGACCGCTGGCAACAAAGGATAAGGGTTGCGCTCGTT
GCGGGACTTAACCCAACATTTCACAACACGAGCTGACGACAGCCATGCAGCACCTGTCTCACGGTTCCCGAAG
GCACATTCTCATCTCTGAAAACTTCCGTGGATGTCAAGACCAGGTAAGGTTCTTCGCGTTGCATCGAATTAAAC
CACATGCTCCACCGCTTGTGCGGGCCCCCGTCAATTCATTTGAGTTTTAACCTTGCGGCCGTACTCCCCAGGCG
GTCGACTTAACGCGTTAGCTCCGGAAGCCACGCCTCAAGGGCACAACCTCCAAGTCGACATCGTTTACGGCGT
GGACTACCAGGGTATCTAATCCTGTTTGCTCCCCACGCTTTCGCACCTGAGCGTCAGTCTTCGTCCAGGGGGCC
GCCTTCGCCACCGGTATTCCTCCAGATCTCTACGCATTTCACCGCTACACCTGGAATTCTACCCCCCTCTACGAG
ACTCAAGCTTGCCAGTATCAGATGCAGTTCCCAGGTTGAGCCCGGGGATTTCACATCTGACTTAACAAACCGC
CTGCGTGCGCTTTACGCCCAGTAATTCCGATTAACGCTTGCACCCTCCGTATTACCGCGGCTGCTGGCACGGA
GTTAGCCGGTGCTTCTTCTGCGGGTAACGTCAATGAGCAAAGGTATTAACTTTACTCCCTTCCTCCCCGCTGAA
AGTACTTTACAACCCGAAGGCCTTCTTCATACACGCGGCATGGCTGCATCAGGCTTGCGCCCATTGTGCAATAT
TCCCCACTGCTGCCTCCCGTAGGAGTCTGGACCGTGTCTCAGTTCCAGTGTGGCTGGTCATCCTCTCAGACCAG
CTAGGGATCGTCGCCTAGGTGAGCCGTTACCCCACCTACTAGCTAATCCCATCTGGGCACATCCGATGGCAAG
AGGCCCGAAGGTCCCCCTCTTTGGTCTTGCGACGTTATGCGGTATTAGCTACCGTTTCCAGTAGTTATCCCCCT
CCATCAGGCAGTTTCCCAGACATTACTCACCCGTCCGCCACTCGTCAGCAAAGAAGCAAGCTTCTTCCTGTTAC
CGTTCGACTTGCATGTGTTAGGCCTGCCGCCAGCGTTCAATCTGAGCCATGATCAAACTCTTCAATTTAAAAGT

TTGATGCTCAAAGAATTAAACTTCGTAATGAATTACGTGTTCACTCTTGAGACTTGGTATTCATTTTTCGTCTTG
CGACGTTAAGAATCCGTATCTTCGAG

>c45470_g1_i1

TCAGATTGAACGCTGGCGGCATGCCTTACACATGCAAGTCGAACGGCAGCGGGGGTAGCTTGCTACCTGCCG
GCGAGTGGCGAACGGGTGAGTAATACATCGGAACGTGCCCTGTAGTGGGGGATAACTAGTCGAAAGACTAG
CTAATACCGCATACGACCTGAGGGTGAAAGTGGGGGACCGCAAGGCCTCATGCTATAGGAGCGGCCGATGTC
TGATTAGCTAGTTGGTGG

>c45470_g1_i2

TCAGATTGAACGCTGGCGGCATGCCTTACACATGCAAGTCGAACGGTAGAGGGGGCAACCCCTTGAGAGTGG
CGAACGGGTGAGTAATGCATCGGAACGTGCCCAGTTGTGGGGGATAACACTTCGAAAGAAGTGCTAATACCG
CATACGACCTGAGGGTGAAAGTGGGGGACCGCAAGGCCTCATGCTATAGGAGCGGCCGATGTCTGATTAGCT
AGTTGGTGG

>c45470_g2_i1

GTTTGATCCTGGCTCAGGACGAACGCTGGCGGCGTGCTTAACACATGCAAGTCGAACGGAAAGGCCCTGCTT
GCAGGGTACTCGAGTGGCGAACGGGTGAGTAACACGTGGGTGATCTGCCCTGCACTTCGGGATAAGCCTGG
GAAACTGGGTCTAATACCGGATAGGAGCCATTTTTAGTGTGATGGTTGGAAAGTTTTTTCGGTGTAGGATGAG
CTCGCGGCCTATCAGCTTGTTGGTGGGGTAATGGCCTACCAAGGCGGCGACGGGTAGCCGGCCTGAGAGGG
TGTA

>c45470_g2_i2

CTCGAGTGGCGAACGGGTGAGTAACACGTGGGTGATCTGCCCTGCACTTCGGGATAAGCCTGGGAAACTGG
GTCTAATACCGGATAGGACCACATTTTGGATGGTGTGGTGGAAAGTTTTTCGGTGTGGGATGAGCTCGCGGC
CTATCAGCTTGTTGGTGAGGTAATGGCTCACCAAGGCGTCGATCCGTAACTGGTCTGAGAGGATGATC

>c46448_g1_i1

CTCCTTGAAAATGAGCTATTCTGTCGGGGTTATTCCACCTCGTCGGAATGGAACATCAAACTTGAGAGTTTGAT
CCTGGCTCAGAACGAACGCTGGCGGCATGCCTAACACATGCAAGTCGAACGATGCTTTCGGGCATAGTGGCG
CACGGGTGCGTAACGCGTGGGAATCTGCCCTTAGGTTCGGAATAACAGTTAGAAATGACTGCTAATACCGGA
TGATGTCGATAAGACCAAAGATTTATCGCCTGAGGATGAGCCCGCGTAGGATTAGCTAGTTGGTGTGGTAAA
GGCGCACCAAGGCGACGATCCTTAGCTGGTCTGAGAGGATGATCAGCCACACTGGGACTGAGACACGGCCCA
GACTCCTACGGGAGGCAGCAG

>c46448_g2_i1

CAGATTGAACGCTGGCGGCAGGCTTAACACATGCAAGTCGAACGATGACTCTCTAGCTTGCTAGAGATGATTA
GTGGCGGACGGGTGAGTAACATTTAGGAATCTGCCTAGTAGTGGGGGATAGCTCGGGGAAACTCGAATTAAT
ACCGCATACGACCTACGGGTGAAAGGGGGCGCAAGCTCTTGCTATTAGATGAGCCTAAATCAGATTAGCTAG
TTGGTGGGGTAAAGGCCCACCAAGGCGACGATCTGTAACTGGTCTGAGAGGATGATCAGTCACACCGGAACT
GAGACACGGTCCGGACTCCTACGGGAGGCAGCA

>c46448_g3_i1

CAGATTGAACGCTGGCGGCAGGCTTAACACATGCAAGTCGAGCGGGGTGATGGTGCTTGCACTATCACTTAG
CGGCGGACGGGTGAGTAATGCTTAGGAATCTGCCTATTAGTGGGGGACAACATCTCGAAAGGGATGCTAATA
CCGCATACGTCCTACGGGAGAAAGCAGGGGATCACTTGTGACCTTGCGCTAATAGATGAGCCTAAGTCGGAT
TAGCTAGTTGGTGGGGTAAAGGCCTACCAAGGCGACGATCTGTAGCGGGTCTGAGAGGATGATCCGCCACAC
TGGGACTGAGACACGGCCCAGACTCCTACGGGAGGCAGCAG

>c46448_g4_i1

GACAATTTAATATTGTGATAAGAAGGGATATGTTGGCGGCGTTTTATTAGTCTGTTTGATATAGGCTAAGGAG
CTGTTGGCGTATTTTTTAATAAACTACGACGTCGGTTTTTTTGTTTTATATTGAGCTGTTAATGTTAGCAGTTTG
ATACTGCTAGGGTTAAACCTGAGAGTTTGATCCTGGCTCAGAGCGAACGCTGGCGGCATGCTTAACACATGCA
AGTCGCACGGACCTTTCGGGGTGAGTGGCGGACGGGTGAGTAACGCGTAGGGATTTATCCATAGGTGGGGG
ATAACACTGGGAAACTGGTGCTAATACCGCATGACACCTGAGGGTCAAAGGCGCGAGTCGCCTATGGAGGA
GCCTGCGTTCGATTAGCTAGTTGGTTAGGTAAAAGCTGACCAAGGCGATGATCGATAGCTGGTCTGAGAGGA
TGATCAGCCACACTGGGACTGAGACACGGCCCAGACTCCTACGGGAGGCAGCAG

>c61060_g1_i1

ACCCTCTCAGGCCGGCTACCCGTCAAAGCCTTGGTAAGCCACTACCCCACCAACAAGCTGATAAGCCGCGAGT
CCATCCCCAACCGCCGAAACTTTCCAACCCCCACCATGCAGCAGGAGCTCCTATCCGGTATTAGCCCCAGTTTC
CTGAAGTTATCCCAAAGTCAAGGGCAGGTTACTCACGTGTTACTCACCCGTTCGCCA

>c67218_g1_i1

GGCCTAACACATGCAAGTCGAACGAACTCTTCGGAGTTAGTGGCGGACGGGTGAGTAACACGTGGGAACGT
GCCTTTAGGTTCGGAATAACTCAGGGAAACTTGTGCTAATACCGAATGTGCCCTTCGGGGGAAAGATTTATCG
CCTTTAGAGCGGCCCGCGTCTGATTAGCTAGTTGGTGAGGTAAAGGCTCACCAAGGCGACGATCAGTAGCTG
GTCTGAGAGGATGATCAG

>c77140_g1_i1

TGGGTGAGTAACACGTGGGGAACCTGCCCCATAGTCTGGGATACCACTTGGAAACAGGTGCTAATACCGGAT
AAGAAAGCAGATCGCATGATCAGCTTTTAAAAGGCGGCGTAAGCTGTCGCTATGGGATGGCCCCGCGGTGCA
TTAGCTAGTTGGTAAGGTAAAGGCTTACCAAGGCGATGATGCATAGCCGAGTTGAGAGACT
