# Supplementary material for: A comparative study of microbial community and dynamics of Asaia in the brown planthopper from susceptible and resistant rice varieties
Source: BMC Microbiol. 2019 Jun 24;19:139. doi: 10.1186/s12866-019-1512-9 (PMC6591912; doi:10.1186/s12866-019-1512-9)
Supplement: Supplementary file 7 — Bacterial sequences of the F6 generation BPHs from the susceptible, TN1, rice variety. (PDF 84 kb) [file 12866_2019_1512_MOESM7_ESM.pdf]

Bacterial sequences of, F6 generation, BPH from resistant, RH, rice variety

>c16130_g1_i1

GGGCAGTAAGCGAATACCTTGCTGTTTTGACGTTACCGACAGAATAAGCACCGGCTAACTCTGTGCCAGCAGC
CGCGGTAATACAGAGGGTGCAAGCGTTAATCGGAATTACTGGGCGTAAAGCGCGCGTAGGTGGCTTGATAA
GTTGGATGTGAAATCCCCGGGCTCAACCTGGGAACTGCATTTGTGACTGCACGGCTAGAGTGTGTCAGAGGG
G

>c22157_g1_i1

TATCTAATCCTGTTTGCTCCCCATGCTTTCGCACCTCAGCGTCAGTGTTAGGCCAGATGGCTGCCTTCGCCATCG
GTATTCCTCCAGATCTCTACGCATTTCACCGCTACACCTGGAATTCTACCATCCTCTCCCATACTCTAGCTTCCCA
GTATCGAATGCAATTCCTAAGTTAAGCTCAGGGATTTCACATCCGACTTAAAAAGCCGCCTACGCACGCTTTAC
GCCCAGTAAATCCGATTAACG

>c22157_g2_i1

GTGATCCAGCCGCAGGTTCCCCTACGGCTACCTTGTTTCGACTTCACCCCAGTCGCTGACCCGACCGTGGTCGG
CTGCGTCCTCGCGGTTCGCTCACCGGCTTCGGGTCAAACCAACTCCCATGGTGTGACGGGCGGTGTGTACAAG
GCCCGGGAACGTATTCACCGCGGCATGCTGATCCGCGATTACTAGCGATTCCACCTTCATGCACTCGAGTTGC
AGAGTACAATCCGAACTGAGACGGCTTTTAGAGATCAGCACGATGTCACCATCTAGCTTCCCACTGTCACCGC
CATTGTAGCACGTGTGTAGCCCAGGTCATAAGGGCCATGAGGACTTGACGTCATCCCCACCTTCCTCCGGCTT
GTCACCGGCAGTTCCTCTAGAGTGCCCACCCAAACATGCTGGCAACTAAAGGCAAGGGTTGCGCTCGTTGCG
GGACTTAACCCAACATCTCACGACACGAGCTGACGACAGCCATGCAGCACCTGTGCTGGAGGTCCCTTGCAG
GAAATATCCATCTCTGAATACAGCCTCCACATGTCAAGACCTGGTAAGGTTCTGCGCGTTGCTTCGAATTAAAC
CACATGCTCCACCGCTTGTGCGGGCCCCCGTCAATTCCTTTGAGTTTCAACCTTGCGGCCGTACTCCCCAGGCG
GTGTGCTTAGCGCGTTAGCTTCGACACTGAAAAACTAAGTTCTCCAACATCCAGCACACATCGTTTACAGCGTG
GACTACCAGGGTATCTAATCCTGTTTGCTCCCCACGCTTTCGCGCCTCAGCGTCAGTAGTGAGCCAGGTTGCCG
CCTTCGCCACCGGTGTTCTTCCCAATATCTACGAATTTCACCTCTACACTGGGAATTCCACAACCCTCTCTCACA
CTCTAGCCTAAACGTATCAAATGCCGTCCCAGGGTTGAGCCCCGGATTTTCACATCTGACTGTCTAAACCGCCT
ACGCGCCCTTTACGCCCAGTCATTCCGAGCAACGCTAGCCCCCTTCGTATTACCGCGGCTGCTGGCACGAAGTT
AGCCGGGGCTTCTTCTACAGGTACCGTCATCATCGTCCCCGTCGAAAGTGCTTTACAATCCGAAGACCTTCTTC
ACACACGCGGCATTGCTGGATCAGGCTTGCGCCCATTGTCCAATATTCCCCACTGCTGCCTCCCGTAGGAGTCT
GGGCCGTGTCTCAGTCCCAGTGTGGCTGATCATCCTCTCAGACCAGCTATCGATCATCGCCTTGGTCAGCTTTT
ACCTAACCAACTAGCTAATCGAACGCAGGCTCCTCCATAGGCGACTCGCGCCTTTGACCCTCAGGTGTCATGC
GGTATTAGCACCAGTTTCCCAGTGTTATCCCCCACCTATGGATAAATCCCTACGCGTTACTCACCCGTCCGCCAC
TCACCCCGAAAGGTCCGTGCGACTTGCATGTGTTAAGCATGCCGCCAGCGTTCGCTCTGAGCCAGGATCAAAC
TCTCAGGTTTAACCCTAGCAGTATCAAACTGCTAACATTAACAGCTCAATATAAAACAAAAAAACCGACGTCGT
AGTTTATTAAAAAATACGCCAACAGCTCCTTAGTCTGTTTGATATAGG

>c22157_g3_i1

CCGTGTCTCAGTCCCAGTGTGGCGGATCATCCTCTCAGACCCGCTACAGATCGTCGCCTTGGTAGGCCTTTACC
CCACCAACTAGCTAATCAGATATCGGCCGCTCCAATAGCGCGAGGTCTTGCGATCCCCCGCTTTCCACCTTAGT
GCGTATGCGGTATTAATCCGGCTTTCGCCGAGCTATCCCCCACTACTGGGTACGTTCCGATATATTACTCACCC
GTTCGCCACTCGCCACCAGGCCGAAGCCCGTGCTGCCGTTCGACTTGCATGTGTAAAGCATGCCGCCAGCGTT
CAATCTGAG

>c22157_g3_i2

CCGTGTCTCAGTCCCAGTGTGGCGGATCATCCTCTCAGACCCGCTACAGATCGTCGCCTTGGTAGGCCTTTACC
CCACCAACTAGCTAATCCGACTTAGGCTCATCTATTAGCGCAAGGTCACAAGTGATCCCCTGCTTTCTCCCGTA
GGACGTATGCGGTATTAGCATCCCTTTCGAGATGTTGTCCCCCACTAATAGGCAGATTCCTAAGCATTACTCAC
CCGTCCGCCGCTAAGTGATAGTGCAAGCACCATCACTCCGCTCGACTTGCATGTGTTAAGCCTGCCGCCAGCG
TTCAATCTGAGCCATGATCAAAC

>c43782_g1_i1

TAAGTCCCGCAACGAGCGCAACCCCTGTTCTATGTTGCCAGCACGGGATGGTGGGGACTCATGGGAGACTGC
CGGGGTCAACTCGGAGGAAGGTGGGGACGACGTCAAATCATCATGCCCCTTATGTCTTGGGCTTCACGCATG
CTACAATGGCCGGTACAATGGGTTGCGATACTGTGAGGTGGAGCTAATCCCTAAAAGCCGGTCTCAGTTCGG
ATTGGGGTCTGCAACTCGACCCCATGAAGTCGGAATCGCTAGTAA
