# Supplementary material for: A comparative study of microbial community and dynamics of Asaia in the brown planthopper from susceptible and resistant rice varieties
Source: BMC Microbiol. 2019 Jun 24;19:139. doi: 10.1186/s12866-019-1512-9 (PMC6591912; doi:10.1186/s12866-019-1512-9)
Supplement: Supplementary file 8 — Bacterial sequences of the F16 generation BPHs from the TN1 rice variety. (PDF 86 kb) [file 12866_2019_1512_MOESM8_ESM.pdf]

Bacterial sequences of, F16 generation, BPH from RH rice variety

>c2877_g1_i1

CAGCCGCGGTAATACGTAGGGTGCGAGCGTTATCCGGAATTACTGGGCGTAAAGAGCTCGTAGGTGGTTTGT
CGCGTCGTCTGTGAAATTCCGGGGCTTAACTCCGGGCGTGCAGGCGATACGGGCATAACTTGAGTGCTGTAG
GGGAGACTGGAATTCCTGGTGTAGCGGTGAAATGCGCAGATATCAGGAGGAACACCGATGGCGAAGGCAGG
TCTCTGGGCTGTTACTGACGCTGAGGAGCGAAAGCATGGGGAGCGAACAGGATTAGATACCCTGGTAG

>c33453_g1_i1

GCAGGCTTAACACATGCAAGTCGAACGATGACTCTCTAGCTTGCTAGAGATGATTAGTGGCGGACGGGTGAG
TAACATTTAGGAATCTGCCTAGTAGTGGGGGATAGCTCGGGGAAACTCGAATTAATACCGCATACGACCTACG
GGTGAAAGGGGGCGCAAGCTCTTGCTATTAGATGAGCCTAAATCAGATTAGCTAGTTGGTGGGGTAAAG

>c37692_g1_i1

TGGCGAACGGGTGAGTAATACGTGAGTAACCTGCCCTTGACTCTGGGATAAGCCTGGGAAACTGGGTCTAAT
ACCGGATAGGAGCCATTTTTAGTGTGATGGTTGGAAAGTTTTTTCGGTGTAGGATGAGCTCGCGGCCTATCAG
CTTGTTGGTGGGGTAATGGCCTACCAAGGCGACGACGGGTAGCCGGCCTGAGAGGGTGACCGGCCACATTG
GGACTGAGATACGGCCCAAACTCCTACGGGAGGCAGCAGTGG

>c37692_g1_i2

AGAGTTTGATCCTGGCTCAGGACGAACGCTGGCGGCGTGCTTAACACATGCAAGTCGAACGGAAAGGCCCTG
CTTTTGTGGGGTGCTCGAGTGGCGAACGGGTGAGTAACACGTGAGTAACCTGCCCTTGACTTTGGGATAACTT
CAGGAAACTGGGGCTAATACCGGATAGGAGCTCCTGCTGCATGGTGGGGGTTGGAAAGTTTCGGCGGTTGG
GGATGGACTCGCGGCTTATCAGCTTGTTGGTGGGGTAGTGGCTTACCAAGGCTTTGACGGGTAGCCGGCCTG
AGAGGGTGACCGGCCACATTGGGACTGAGATACGGCCCAAACTCCTACGGGAGGCAGCAGTGG

>c39174_g2_i1

ACCGCTACACCTGGAATTCTACCATCCTCTCCCACACTCTAGCCAACCAGTATCGAATGCAATTCCCAAGTTAA
GCTCGGGGATTTCACATTTGACTTAATTGGCCGCCTACGCGCGCTTTACGCCCAGTAAATCCGATTAACGCTTG
CACCCTCTGTATTACCGCGGCTGCTGGCACAGAGTTAGCCGGTGCTTATTCTGCGAGTAACGTCCACTCATCTT
GGGTATTAACCAAAAGAGCCTCCTCCTCGCTTAAAGTGCTTTACAACCATAAGGCCTTCTTCACACACGCGGCA
TGGCTGGATCAGGGTTCCCCCCATTGTCCAATATTCCCCACTG

>c39368_g1_i1

GTGCCAGCAGCCGCGGTAATACGAAGGGGGCTAGCGTTGCTCGGAATGACTGGGCGTAAAGGGCGCGTAGG
CGGTTTAGACAGTCAGATGTGAAAATCCGGGGCTCAACCCTGGGACGGCATTTGATACGTTTAGGCTAGAGT
GTGAGAGAGGGTTGTGGAATTCCCAGTGTAGAGGTGAAATTCGTAGATATTGGGAAGAACACCGGTGGCGA
AGGCGGCAACCTGGCTCACTACTGACGCTGAGGCGCGAAAGCGTGGGGAGCAAACAG

>c45170_g1_i1

CAGATTGAACGCTGGCGGCAGGCTTAACACATGCAAGTCGAGCGGGGTGATGGTGCTTGCACTATCACTTAG
CGGCGGACGGGTGAGTAATGCTTAGGAATCTGCCTATTAGTGGGGGACAACATCTCGAAAGGGATGCTAATA
CCGCATACGTCCTACGGGAGAAAGCAGGGGATCACTTGTGACCTTGCGCTAATAGATGAGCCTAAGTCGGAT

TAGCTAGTTGGTGGGGTAAAGGCCTACCAAGGCGACGATCTGTAGCGGGTCTGAGAGGATGATCCGCCACAC
TGGGACTGAGACACGGCCCAGACTCCTACGGGAGGCAGCAG

>c45170_g2_i1

TCGGCTAACAATGGTGGGACCTCGTCAAACGTTGTGATCAGCCGGTTTAAAGTTTCAAGTCCAACTTGAGAGT
TTGATCCTGGCTCAGAGCGAACGCTGGCGGCATGCTTAACACATGCAAGTCGCACGGACCTTTCGGGGTGAG
TGGCGGACGGGTGAGTAACGCGTAGGGATTTATCCATAGGTGGGGGATAACACTGGGAAACTGGTGCTAAT
ACCGCATGACACCTGAGGGTCAAAGGCGCGAGTCGCCTATGGAGGAGCCTGCGTTCGATTAGCTAGTTGGTT
AGGTAAAAGCTGACCAAGGCGATGATCGATAGCTGGTCTGAGAGGATGATCAGCCACACTGGGACTGAGAC
ACGGCCCAGACTCCTACGGGAGGCAGCAG

>c45170_g3_i1

TGAACGCTGGCGGCATGCCTAACACATGCAAGTCGAACGATTGCCTTCGGGTGATAGTGGCGCACGGGTGCG
TAACGCGTGGGAATCTGCCCTTTGGTTCGGAATAACAGTTGGAAACGACTGCTAATACCGGATGATGACGTTA
AGTCCAAAGATTTATCGCCGAGGGATGAGCCCGCGTAGGATTAGCTAGTTGGTGTGGTAAAGGCGCACCAAG
GCGACGATCCTTAGCTGGTCTGAGAGGATGATCAGCCACACTGGGACTGAGACACGGCCCAGACTCCTACGG
GAGGCAGCAG

>c45513_g1_i1

CGAGGTGATCCAACCGCAGGTTCCCCTACGGTTACCTTGTTACGACTTCACCCCAGTCATGAATCACAAAGTG
GTAAGCGCCCTCCCGAAGGTTAAGCTACCTACTTCTTTTGCAACCCACTCCCATGGTGTGACGGGCGGTGTGT
ACAAGGCCCGGGAACGTATTCACCGTGGCATTCTGATCCACGATTACTAGCGATTCCGACTTCATGGAGTCGA
GTTGCAGACTCCAATCCGGACTACGACGCACTTTATGAGGTCCGCTTGCTCTCGCGAGGTCGCTTCTCTTTGTA
TGCGCCATTGTAGCACGTGTGTAGCCCTGGTCGTAAGGGCCATGATGACTTGACGTCATCCCCACCTTCCTCCA
GTTTATCACTGGCAGTCTCCTTTGAGTTCCCGGCCGGACCGCTGGCAACAAAGGATAAGGGTTGCGCTCGTTG
CGGGACTTAACCCAACATTTCACAACACGAGCTGACGACAGCCATGCAGCACCTGTCTCACGGTTCCCGAAGG
CACATTCTCATCTCTGAAAACTTCCGTGGATGTCAAGACCAGGTAAGGTTCTTCGCGTTGCATCGAATTAAACC
ACATGCTCCACCGCTTGTGCGGGCCCCCGTCAATTCATTTGAGTTTTAACCTTGCGGCCGTACTCCCCAGGCGG
TCGACTTAACGCGTTAGCTCCGGAAGCCACGCCTCAAGGGCACAACCTCCAAGTCGACATCGTTTACGGCGTG
GACTACCAGGGTATCTAATCCTGTTTGCTCCCCACGCTTTCGCACCTGAGCGTCAGTCTTCGTCCAGGGGGCCG
CCTTCGCCACCGGTATTCCTCCAGATCTCTACGCATTTCACCGCTACACCTGGAATTCTACCCCCCTCTACGAGA
CTCAAGCTTGCCAGTATCAGATGCAGTTCCCAGGTTGAGCCCGGGGATTTCACATCTGACTTAACAAACCGCCT
GCGTGCGCTTTACGCCCAGTAATTCCGATTAACGCTTGCACCCTCCGTATTACCGCGGCTGCTGGCACGGAGTT
AGCCGGTGCTTCTTCTGCGGGTAACGTCAATGAGCAAAGGTATTAACTTTACTCCCTTCCTCCCCGCTGAAAGT
ACTTTACAACCCGAAGGCCTTCTTCATACACGCGGCATGGCTGCATCAGGCTTGCGCCCATTGTGCAATATTCC
CCACTGCTGCCTCCCGTAGGAGTCTGGACCGTGTCTCAGTTCCAGTGTGGCTGGTCATCCTCTCAGACCAGCTA
GGGATCGTCGCCTAGGTGAGCCGTTACCCCACCTACTAGCTAATCCCATCTGGGCACATCTGATGGCAAGAGG
CCCGAAGGTCCCCCTCTTTGGTCTTGCGACGTTATGCGGTATTAGCTACCGTTTCCAGTAGTTATCCCCCTCCAT
CAGGCAGTTTCCCAGACATTACTCACCCGTCCGCCACTCGTCAGCAAAGAAGCAAGCTTCTTCCTGTTACCGTT
CGACTTGCATGTGTTAGGCCTGCCGCCAGCGTTCAATCTGAGCCATGATCAAACTCTTCAATTTAAAAGTTTGA
TGCTCAAAGAATTAAACTTCGTAATGAATTACGTGTTCACTCTTGAGACTTGGTATTCATTTTTCGTCTTGCGAC
GTTAAGAATCCGTATCTTCGAG

>c50751_g1_i1

GGCCTAACACATGCAAGTCGAACGAACTCTTCGGAGTTAGTGGCGGACGGGTGAGTAACACGTGGGAACGT
GCCTTTAGGTTCGGAATAACTCAGGGAAACTTGTGCTAATACCGAATGTGCCCTTCGGGGGAAAGATTTATCG
CCTTTAGAGCGGCCCGCGTCTGATTAGCTAGTTGGTGAGGTAATGGCTCACCAAGGCGACGATCAGTAGCTG
GTCTGAGAGGATGATCAG

>c53891_g1_i1

AAAAGAACGTAGTTAACGACTATCGTTAGATCATAGCATACGGGTGAGTTTTATATAGGAATATAAACTAATA
TATGGAAAAGGAATAAATAAGGGGAAACCCGCCATAAAATAGAGCCTATAAAAAAGATTAGGTAGTTGGTAA
GGTAATGGCTTACCAAGCCGAGGATCTGAAATCTATACTAGAAAAAAGATAGATCACAG
