# Supplementary material for: A comparative study of microbial community and dynamics of Asaia in the brown planthopper from susceptible and resistant rice varieties
Source: BMC Microbiol. 2019 Jun 24;19:139. doi: 10.1186/s12866-019-1512-9 (PMC6591912; doi:10.1186/s12866-019-1512-9)
Supplement: Supplementary file 12 — Bacterial sequences of the F16 generation BPHs from the RH rice variety. (PDF 87 kb) [file 12866_2019_1512_MOESM12_ESM.pdf]

Bacterial sequences of, F0 generation, BPH

>c20088_g1_i1

AGTCCCGCAACGAGCGCAACCCTTTTCCTTACTTGCCAGCATTTCGGATGGGAACTTTAAGGATACTGCCAGTG
ACAAACTGGAGGAAGGCGGGGACGACGTCAAGTCATCATGGCCCTTACGGCCAGGGCTACACACGTGCTACA
ATGGTCGGTACAAAGGGTTGCTACCTAGCGATAGGATGCTAATCTCAAAAAGCCGATCGTAGTCCGGATTGG
AGTCTGCAACTCGACTCCATGAAGTCGGAATCGCTAGTAATCGTAGATCAGAATGCTACGGTGAATACGTTCC
CGGGCCTTGTACACACCGCCCGTCACACCATGGGAG

>c27627_g1_i1

CTTTCACCGATGAAGATAATGACGGTAGTCGGAGAAGAAGCCCCGGCTAACTTCGTGCCAGCAGCCGCGGTA
ATACGAAGGGGGCTAGCGTTGTTCGGATTTACTGGGCGTAAAGCGCACGTAGGCGGACTTTTAAGTCAGGGG
TGAAATCCCAGAGCTCAACTCTGGAACTGCCTTTGATACTGGAAGTCTTGAGTATGGTAGAGGTGAGTGGAAT
TCCGAGTGTAGAGGTGAAATTCGTAGATATTCGGAGGAACACCAGTGGCGAAGGCGGCTCACTGGACCATTA
CTGACGCTGAGGTGCGAAAGCGTGGGGAGCAAACAGGATTAGATACCCTGGTAGTCCACGCCGTAAACGAT
GTCAACTAGCCGTTGGAATCCTTGAGATTTTAGTGGCGCAGCTAACGCATTAAGTTGACCGCCTGGGGAGTAC
GGCCGCAAGGTTAAAACTCAAATGAATTGACGGGGGCCCGCACAAGCGGTGGAGCATGTGGTTTAATTCGAT
GCAACGCGAAGAACCTTACCTGGCCTTGACATAGTAGAAACTTTCCAGAGATGGATTGGTGCCTTCGGGAATC
TACATACAGGTGCTGCATGGCTGTCGTCAGCTCGTGTCGTGAGATGTTGGGTTAAGTCCCGCAACGAGCGCAA
CCCTTACGATTAGTTGCTACGCAAGAGCACTCTAATAGGACTGCCGTTGACAAAACGGAGGAAGGTGGGGAT
GACGTCAAGTCCTCATGGCCCTTATGACCTGGGCTACACACGTGCTACAATGGCGGTGACAAATGGCCGGAA
ACCCGCGGGGGGGGGCTAATTTC

>c27627_g1_i2

GGGAACCCTGATCCAGCCATGCCGCGTGTGTGAAGAAGGCCTTATGGTTGTAAAGCACTTTAAGCGAGGAGG
AGGCTCTTTTGGTTAATACCCAAGATGAGTGGACGTTACTCGCAGAATAAGCACCGGCTAACTCTGTGCCAGC
AGCCGCGGTAATACAGAGGGTGCAAGCGTTAATCGGATTTACTGGGCGTAAAGCGCGCGTAGGCGGCCAATT
AAGTCAAATGTGAAATCCCCGAGCTTAACTTGGGAATTGCATTCGATACTGGTTGGCTAGAGTGTGGGAGAG
GATGGTAGAATTCCAGGTGTAGCGGTGAAATGCGTAGAGATCTGGAGGAATACCGATGGCGAAGGCAGCCA
TCTGGCCTAACACTGACGCTGAGGTGCGAAAGCATGGGGAGCAAACAGGATTAGATACCCTGGTAGTCCATG
CCGTAAACGATGTCTACTAGCCGTTGGGGCCTTTGAGGCTTTAGTGGCGCAGCTAACGCGATAAGTAGACCGC
CTGGGGAGTACGGTCGCAAGACTAAAACTCAAATGAATTGACGGGGGCCCGCACAAGCGGTGGAGCATGTG
GTTTAATTCGATGCAACGCGAAGAACCTTACCTGGCCTTGACATAGTAGAAACTTTCCAGAGATGGATTGGTG
CCTTCGGGAATCTACATACAGGTGCTGCATGGCTGTCGTCAGCTCGTGTCGTGAGATGTTGGGTTAAGTCCCG
CAACGAGCGCAACCCTTACGATTAGTTGCTACGCAAGAGCACTCTAATAGGACTGCCGTTGACAAAACGGAG
GAAGGTGGGGATGACGTCAAGTCCTCATGGCCCTTATGACCTGGGCTACACACGTGCTACAATGGCGGTGAC
AAATGGCCGGAAACCCGCGGGGGGGGGCTAATTTC

>c27627_g1_i3

GAAGGCCTTCGGGTTGTAAAGTACTTTCAGCGGGGAGGAAGGGTTGAGTGTTAATATCACTCAGCATTGACG
TTACCCGCAGAAGAAGCACCGGCTAACTCCGTGCCAGCAGCCGCGGTAATACGGAGGGTGCAAGCGTTAATC
GGAATTACTGGGCGTAAAGCGCACGCAGGCGGTTTGTTAAGTCAGATGTGAAATCCCCGGGCTCAACCTGGG
AACTGCATTTGAAACTGGCAAGCTAGAGTCTCGTAGAGGGGGGTAGAATTCCAGGTGTAGCGGTGAAATGCG
TAGAGATCTGGAGGAATACCGGTGGCGAAGGCGGCCCCCTGGACGAAGACTGACGCTCAGGTGCGAAAGCG

TGGGGAGCAAACAGGATTAGATACCCTGGTAGTCCACGCCGTAAACGATGTCAACTAGCCGTTGGAATCCTT
GAGATTTTAGTGGCGCAGCTAACGCATTAAGTTGACCGCCTGGGGAGTACGGCCGCAAGGTTGAAACTCAAA
G

>c27627_g2_i1

TGTTATTAGGGAAGAACAAACGTGTAAGTAACTGTGCACGTCTTGACGGTACCTAATCAGAAAGCCACGGCTA
ACTACGTGCCAGCAGCCGCGGTAATACGTAGGGTGCAAGCGTTGTCCGGAATTATTGGGCGTAAAGAGCTCG
TAGGCGGCTTGTCGCGTCTGCTGTGAAAACCCGAGGCTCAACCTCGGGCCTGCAGTGGGTACGGGCAAGCTA
GAGTGCGGTAGGGGAGATTGGAATTCCTGGTGTAGCGGTGGAATGCGCAGATATCAGGAGGAACACCGATG
GCGAAGGCAGATCTCTGGGCCGTAACTGACGCTGAGGAGCGAAAGCATGGGGAGCGAACAGGATTAGATAC
CCTGGTAGTCCATGCCGTAAACGATGTCTACTAGCCGTTGGGGCCTTTGAGGCTTTAGTGGCGCAGCTAACGC
GATAAGTAGACCGCCTGGGGAGTACGGTCGCAAGACTGAAACTCAAAG

>c27627_g3_i1

CGGGTGCTAATACTGGATATTCACTGATCTTCGCATGGGGGTTGGTGGAAAGGTTTTTTCTGGTGGGGGATG
GGCTCGCGGCCTATCAGCTTGTTGGTGAGGTAATGGCTCACCAAGGCGACGACGGGTAGCCGGCCTGAGAG
GGTGACCGGCCACACTGGGACTGAGACACGGCCCAGACTCCTACGGGAGGCAGCAGTGGGGAATATTGCAC
AATGGGCGCAAGCCTGATG

>c27627_g4_i1

TTGAGTCAACAAATTAAATCTATTGACACCGCGTAGCGATGACAATAGATAGAAAGATTAAACTGAAGAGTTT
GATCATGGCTCAGATTGAACGCTGGCGGCAGGCTTAACACATGCAAGTCGAGCGGTAGCACAGGGGAGCTT
GCTCCCTGGGTGACGAGCGGCGGACGGGTGAGTAATGTCTGGGAAACTGCCTGATGGAGGGGGATAACTAC
TGGAAACGGTAGCTAATACCGCATAACGTCGCAAGACCAAAGAGGGGGACCTTCGGGCCTCTTGCCATCAGA
TGTGCCCAGATGGGATTAGCTAGTAGGTGGGGTAATGGCTCACCTAGGCGACGATCCCTAGCTGGTCTGAGA
GGATGACCAGCCACACTGGAACTGAGACACGGTCCAGACTCCTACGGGAGGCAGCAGTGGGGAATATTGCA
CAATGGGCGCAAGCCTGATG

>c48723_g1_i1

ACGAGTGGCGAACGGGTGAGTAATACATCGGAACGTGCCCAGTCGTGGGGGATAACTACTCGAAAGAGTAG
CTAATACCGCATACGATCTGAGGATGAAAGCGGGGGACCTTCGGGCCTCGCGCGATTGGAGCGGCCGATGG
CAGATTAGGTAGTTGGTGGGATAAAAGCTTACCAAGCCGACGATCTGTAGCTGGTCTGAGAGGACGACCAGC
CACACTGGGACTGAGACACG

>c55974_g1_i1

AAAAAAAAAAAAAAAAATAAAAAAGGCAATAAATAATAATTGCAAAAAAACTAAAAATAGTTTGATCTTGGCT
ACGAATTTACGCTAACTATCGGCATTACACATGCAAGTTGTATGAAAAGAACGTAGTTAACGACTATCGTTAG
ATCATAGCATACGGGTGAGTTTTATATAGGAATATAAACTAATATATGGAAAAGGAATAAATAAGGGGAAAC
CCGCCATAAAATAGAGCCTATAAAAAAGATTAGGTAGTTGGTAAGGTAATGGCTTACCAAGCCGAGGATCTG
AAATCTATACTAGAAAAAAGATAGATCACAGTAGCAATGAAAAAGAGCTACGTAGGATAAATAAGCCTGCCA
GCAGTGGGGAATCTTGGACAATGGGGGAAACCCTGATCCAGCAAGATAGTAAGAGTGCAAAGTAAATAAGC
ATAAAACTTCTAAAGAGAAAGAAAAATAATGATATAACTTCTCAATTAAATCCTGACAAATTTCGTGCCAGCAG
TCGCGGTTATACGAAAAGGATGAGCGTAATTCATAATATCTAGGTGTAAAGGGTCAGTAGGCAGCTAATTTG
AATCAAAACTAAAAGTGTTGATTCTATGTTTTTAAACAGAAATAAATTAGCTTGAGTGTATAAAGGGGAATAG

AAATTAGAGGTGTAGGGATAGAATCTTATTATATTTCTAGGAATACTAAAAAGCGAAGGCATTTTTCTTTGAA
ACACTGACGCTAATAGACTAAAGTGTGGGTAGCGAAGAGGATTAGATACCCTATTAGTCCACACCCTAAACGA
TGAGTGCTATATTAAACAAGAAAGCATTCCACCTGGGGAGTACATTCGCAAGAAAGAAACTTAAAAGAATAG
ACGGTTTTTAAGACCAGCAGTGAAGCATGTTATTTAATTTGATAGCACCCAAATAATCTTACCAATCTTTGAAT
AGATTATTAGATATATTGAAAAGCATAACAGCTCTTGTTTAGGCAAGTGCAGTATATGAATGGAAATATATTT
GTAATTTACAGGTATTGCATGGCTGTCTTCAGTTCGTGTTTTGAGATGTATGGTTAGGTCCGAGAACGAACGA
AATCCTCTTTACTAATTAAATCTTAGTAAAATCGACTCCTAAAGGAAAGATCGAGGGGGTTAAAGACAAGTCC
TTATGGTTTTAATAGATTGGGCTATAGACGTGCCACATAGGTAGTAACAATAGGAAATGAAGAAAACCTTAAA
AACTACTATGTACAGATGGAAGTCTGAAATTCGACAACCTGAAGTAGGAATTGCTAGTAATCGTGAATCATGA
TGTCACGGTGAAAAAGAATATTAACTATGTACTAATCGCCCGTCAAGGATAGGGA

>c58357_g1_i1

GTCTCAGTACCAGTGTGGGGGATCACCCTCTCAGGCCCCCTACCCATCGTAGCCTTGGTGTGCCGTTACCACAC
CAACTAGCTAATGGGACGCATGCCCATCCTATACCGTAACCTTTAATCAATAAGTGATGCCACTCATTAACACC
ATGGGGTATTAGTCCGAATTTCTCCGGGATATCCCCCAGTATAGGGTAGGTTGCATACGCGTTACGCACCCGT
GCGCCGGTCGCCA
