# Supplementary material for: A comparative study of microbial community and dynamics of Asaia in the brown planthopper from susceptible and resistant rice varieties
Source: BMC Microbiol. 2019 Jun 24;19:139. doi: 10.1186/s12866-019-1512-9 (PMC6591912; doi:10.1186/s12866-019-1512-9)
Supplement: Supplementary file 13 — Asaia-specific primer design. Asaia-specific 16S rRNA gene sequences belonging to the NCBI Accession Numbers NR_122089.1, NR_112880.1, NR_024810.1, NR_114144.1, NR_041564.1, NR_113849.1, NR_024728.1, NR_112879.1, NR_112953.1, NR_113845.1, and NR_024738.1 were used. (PDF 85 kb) [file 12866_2019_1512_MOESM13_ESM.pdf]

Bacterial sequences of, F6 generation, BPH from susceptible, TN1, rice variety

>c17110_g1_i1

GCCTCCCGTAGGAGTCTGGGCCGTATCTCAGTCCCAATGTGGCCGTCCACCCTCTCAGGCCGGCTACCCGTCG
CCGCCTTGGTAGGCCATTACCCCACCAACAAGCTGATAGGCCGCGAGCTCATCCTACACCGAAAAAACTTTCC
AACCATCACACTAAAAATGGCTCCTATCCGGTATTAGACCCAGTTTCCCAGGCTTATCCCGAAGTGCAGGGCA
GATCACCCACGTGTTACTCACCCGTTCGCCACTCGAGTACCCTGCAAGCAGGGCCTTTCCGTTCGACTTGCAAG
TGTTAAGCACGCCGCCAG

>c21876_g1_i1

TGTTTGGCTAGAGTGTGGGAGAGGATGGTAGAATTCCAGGTGTAGCGGTGAAATGCGTAGAGATCTGGAGG
AATACCGATGGCGAAGGCAGCTTCCTGGCATAATATTGACACTGAGATTCGAAAGCGTGGGTAGCAAACAGG
ATTAGATACCCTGGTAGTCCACGCTGTAAACGATGTGTGCTGGATGTTGGAGAACTTAGTTTTTCAGTGTCGA
AGCTAACGCGCTAAGCACACCGCCTGGGGAGTACGGCCGCAAGGTTGAAACTCAAAGGAATTGACGGGGGC
CCGCACAAGCGGTGGAGCATGTGGTTTAATTCGAAGCAACACG

>c21876_g1_i2

ATTCAAAGTTGGTGATTCGTCGAGTCAAGATATACCGAAGGTTTCGGCCTTCAAGAAAACTTAAACTGAAGAG
TTTGATCATGGCTCAGATTGAACGCTGGCGGCAGGCTTAACACATGCAAGTCGAGCGGAGTGATGGTGCTTG
CACTATCACTTAGCGGCGGACGGGTGAGTAATGCTTAGGAATCTGCCTATTAGTGGGGGACAACATCTCGAA
AGGGATGCTAATACCGCATACGTCCTACGGGAGAAAGCAGGGGATCACTTGTGACCTTGCGCTAATAGATGA
GCCTAAGTCGGCTTAGCTAGTTGGTGGGGTAAAGGCCTACCAAGGCGACGATCTGTAGCGGGTCTGAGAGG
ATGATCCGCCACACTGGGACTGAGACACGGCCCAGACTCCTACGGGAGGCAGCAGTGGGGAATATTGGACA
ATGGGCGCAAGCCTGATCCAGCAATGCCGCGTGTGTGAAGAAGGTCTTCGGATTGTAAAGCACTTTCGACGG
GGACGATGATGACGGTACCTGTAGAAGAAGCCCCGGCTAACTTCGTGCCAGCAGCCGCGGTAATACGAAGG
GGGCTAGCGTTGCTCGGAATGACTGGGCGTAAAGGGCGCGTAGGCGGTTTAGACAGTCAGATGTGAAAATC
CGGGGCTCAACCCTGGGACGGCATTTGATACGTTTAGGCTAGAGTGTGAGAGAGGGTTGTGGAATTCCCAGT
GTAGAGGTGAAATTCGTAGATATTGGGAAGAACACCGGTGGCGAAGGCGGCAACCTGGCTCACTACTGACG
CTGAGGCGCGAAAGCGTGGGGAGCAAACAGGATTAGATACCCTGGTAGTCCACGCTGTAAACGATGTGTGCT
GGATGTTGGAGAACTTAGTTTTTCAGTGTCGAAGCTAACGCGCTAAGCACACCGCCTGGGGAGTACGGCCGC
AAGGTTGAAACTCAAAGGAATTGACGGGGGCCCGCACAAGCGGTGGAGCATGTGGTTTAATTCGAAGCAAC
ACG

>c21876_g1_i3

GTTTGATCCTGGCTCAGAGCGAACGCTGGCGGCATGCTTAACACATGCAAGTCGCACGGACCTTTCGGGGTG
AGTGGCGGACGGGTGAGTAACGCGTAGGGATTTATCCATAGGTGGGGGATAACACTGGGAAACTGGTGCTA
ATACCGCATGACACCTGAGGGTCAAAGGCGCGAGTCGCCTATGGAGGAGCCTGCGTTCGATTAGCTAGTTGG
TTAGGTAAAAGCTGACCAAGGCGATGATCGATAGCTGGTCTGAGAGGATGATCAGCCACACTGGGACTGAGA
CACGGCCCAGACTCCTACGGGAGGCAGCAGTGGGGAATATTGGACAATGGGCGCAAGCCTGATCCAGCAAT
GCCGCGTGTGTGAAGAAGGTCTTCGGATTGTAAAGCACTTTCGACGGGGACGATGATGACGGTACCTGTAGA
AGAAGCCCCGGCTAACTTCGTGCCAGCAGCCGCGGTAATACGAAGGGGGCTAGCGTTGCTCGGAATGACTGG
GCGTAAAGGGCGCGTAGGCGGTTTAGACAGTCAGATGTGAAAATCCGGGGCTCAACCCTGGGACGGCATTT
GATACGTTTAGGCTAGAGTGTGAGAGAGGGTTGTGGAATTCCCAGTGTAGAGGTGAAATTCGTAGATATTGG
GAAGAACACCGGTGGCGAAGGCGGCAACCTGGCTCACTACTGACGCTGAGGCGCGAAAGCGTGGGGAGCA

AACAGGATTAGATACCCTGGTAGTCCACGCTGTAAACGATGTGTGCTGGATGTTGGAGAACTTAGTTTTTCAG
TGTCGAAGCTAACGCGCTAAGCACACCGCCTGGGGAGTACGGCCGCAAGGTTGAAACTCAAAGGAATTGACG
GGGGCCCGCACAAGCGGTGGAGCATGTGGTTTAATTCGAAGCAACACG

>c33632_g1_i1

GACCAATGATCACCCATGACTTCTGTGTACCACTAGTGCCACAAAGGCTTTTGGTGGTCTTATTGGCGCATACC
ACGGTGGGGTCTTCGACTGGGGTGAAGTCGTAACAAGGTAGCCGTAGGGGAACCTGTGGCTGGATTGAATC
CTTCGCGATGGAAATGCCCTCTTCCCCCAACCCAAACTCAACAAAGGGCAAGCACCTACTCCTAACAAAATGA
AAAGCA

>c44959_g1_i1

TGTGTGTACAAGGCCCGGGAACGTATTCACCGCAGCGTTGCTGATCTGCGATTACTAGCGACTCCGACTTCAT
GGGGTCGAGTTGCAGACCCCAATCCGAACTGAGACCGGTTTTAAGAGATTCGCTCCGCCTCACGGCATCGCA
GCCCTCTGTACCGGCCATTGTAGCATGTGTGAAGCCCTGGACATAAGGGGCATGATGATTTGACGTCATCCCC
ACCTTCCTCCGGTTTGTCACCGGCAGTCAACTTAGAGTGCCCAACTTAATGATGGCAACTAA

>c53346_g1_i1

CTGCGTTGTATTAGCTAGTTGGTGAGGTAACGGCTCACCAAGGCAGTGATACGTAGCCGACCTGAGAGGGTA
ATCGGCCACATTGGGACTGAGACACGGCCCAAACTCCTACGGGAGGCAGCAGTAGGGAATCTTCCGCAATGG
GCGAAAGCCTGACGGAGCAATGCCGCGTGGAGGTAGAAGGCCTACGGGTCGTGAACTTCTTTTCCCGGAGAA
GAAGCAATGACGGTATCTGG

>c61338_g1_i1

TGAACTAATACTTTGGGCTAATGACGGTACCTGAAGAATAAGCACCGGCTAACTACGTTCCAGCAGCCGCGGT
AATACGTAGGTGGCAAGCGTTGTCCGGATTTATTGGGCGTAAAGGGAGCGCAGGCGGTGACTTAAGTCTGAT
GTGAAAGCCCACGGCTCAACCGTGGAGGGTCATTGGAAACTGGGGAACTTGAGTGCAGAAGAGGAG

>c8693_g1_i1

GGGCGTAAAGGGCTCGTAGGTGGTTTGTCGCGTCGTCTGTGAAATTCCGGGGCTTAACTCCGGGCGTGCAGG
CGATACGGGCATAACTTGAGTGCTGTAGGGGAGACTGGAATTCCTGGTGTAGCGGTGAAATGCGCAGATATC
AGGAGGAACACCGATGGCGAAGGCAGGTCTCTGGGCAGTAACTGACGCTGAGGAGCGAAAGCATGG

>c9803_g1_i1

ATTGACGGGGGCCCGCACAAGCGGCGGAGCATGCGGATTAATTCGATGCAACGCGAAGAACCTTACCAAGG
CTTGACATGTTCTCGATCGCCGTAGAGATACGGTTTCCCCTTTGGGGCGGGTTCACAGGTGGTGCATGGTTGT
CGTCAGCTCGTGTCGTGAGATGTTGGGTTAAGTCCCGCAACGAGCGCAACCCTTGCCTTTAGTTGCCAGCATG
TTTGGGTGGGCACTCTAGAGGAACTGCCGGTGACAAGCCGGAGGAAGGTGGGGATGACGTC
